# Scalable differentiation of human cardiac organoids from iPSCs generates cardiac tissues for cardiac cell therapy

**DOI:** 10.7150/thno.127654

**Published:** 2026-04-23

**Authors:** Sarkawt Hamad, Ebru Aksoy, Maria Kalil, Daina Martínez-Falguera, Christian Jüngst, Katharina Zahn, Felix Gaedke, Gabriel Peinkofer, Bastiaan J Boukens, Rezwan Firuzi, Rebecca Prause, Julia Aranyo, Felipe Bisbal, Oriol Iborra-Egea, Esther Jorge, Georgina Iraola-Picornell, Albert Teis, Martina de Raffele, Victoria Delgado, Frank Nitsche, Agapios Sachinidis, Markus Khalil, Fatma Alhashimi, Astrid Schauss, Antoni Bayes-Genis, Carolina Gálvez-Montón, Kurt P Pfannkuche

**Affiliations:** 1 Center for Physiology and Pathophysiology, Physiology I, University and University Hospital of Cologne, Cologne, Germany.; 2 Biology Department, Faculty of Science, Soran University, Soran, Kurdistan Region-Iraq.; 3 Marga and Walter Boll-Laboratory for Cardiac Tissue Engineering, University of Cologne, Cologne, Germany.; 4 ICREC Research Program, Germans Trias i Pujol Research Institute (IGTP), Badalona, Barcelona, Spain.; 5 Department of Medicine, Universitat Autònoma de Barcelona (UAB), Barcelona, Spain.; 6 Department of Medicine, Universitat de Barcelona (UB), Barcelona, Spain.; 7 Cologne Excellence Cluster on Cellular Stress Responses in Aging-Associated Diseases (CECAD), Imaging Facility, Cologne, Germany.; 8 Department of Medical Biology, Amsterdam University Medical Center, Amsterdam, The Netherlands.; 9 Department Physiology, University Maastricht, Maastricht University Medical Center, Maastricht, The Netherlands.; 10 Environmental Monitoring and Endocrinology, Faculty of Biology, Technische Universität Dresden, Dresden, Germany.; 11 Heart Institute (iCOR), Germans Trias i Pujol University Hospital, Badalona, Barcelona, Spain.; 12 CIBER Cardiovascular, Instituto de Salud Carlos III, Madrid, Spain.; 13 Department of Basic Sciences, Faculty of Health Sciences at Manresa, University of Vic - Central University of Catalonia (UVic-UCC), Manresa, Spain.; 14 University of Cologne, Institute for Zoology, General Ecology, Cologne, Germany.; 15 Department of Paediatric Cardiology, University Hospital of Cologne, Cologne, Germany.; 16 First Stem Cell and Genomics Laboratory, Dubai, UAE.; 17 Center for Molecular Medicine (CMMC), Cologne, Germany.

**Keywords:** hiPSC, cardiac organoids, cardiac cell therapy, myocardial infarction

## Abstract

**Rationale:**

Cardiac organoids have the potential to overcome the limitations of cell-based therapies for myocardial infarction, particularly by improving cell engraftment.

**Methods:**

Differentiation of iPSCs into COs with matured-like action potentials and sarcomere lengths was achieved within 20 days. COs comprised cardiomyocytes, cardiac fibroblasts and endothelial cells. Transmission electron microscopy and light microscopy revealed organized sarcomeres and the presence of potential gap junctions. Furthermore, we validate a bioreactor-assisted upscaling strategy to produce the high cell densities necessary for cardiac regenerative medicine. The transplantation of COs into acutely infarcted pig hearts was successfully achieved, serving as a preclinical model for cardiac cell therapy.

**Results:**

A modest quantity of 3,500 organoids was used, and notable levels of engraftment into the infarcted area of the pig's heart were observed. In the 8- to 30-day follow-up period, the administration of CO did not result in the occurrence of arrhythmic events. Furthermore, CO-treated pigs exhibited an enhancement in cardiac function and a reduction in scar size when compared to the control group. Spatial transcriptomics demonstrates the downregulation of pro-fibrotic signalling in day 30 implanted COs.

**Conclusions:**

In summary, this study presents a novel technology for generating cardiac organoids composed of the primary cell types of functional myocardium, offering a platform for developing cell-based heart therapies.

## Introduction

The mammalian heart is the first organ to develop and is essential for maintaining systemic circulation and oxygen exchange across tissues 1. Structurally, it is composed predominantly of cardiomyocytes (CMs), supported by endothelial cells (ECs), smooth muscle cells (SMCs), cardiac fibroblasts (CFs ; cFB), and extracellular matrix (ECM) 2, 3. The mammalian heart forms a compact wall during foetal development, allowing high blood pressures and fast filling of the chambers. The open-chamber structure is a hallmark of the superior performance of mammalian hearts in support of high metabolic rates in the body.

In the aftermath of a myocardial infarction (MI) occasioned by coronary artery occlusion, a substantial number of cardiomyocytes perish. These are subsequently replaced by fibrotic scar tissue, which is characterized by a high collagen type I content 4-6. While the scar formation is of vital importance to stabilize the wall and prevent ventricular rupture, it compromises contractile function, often leading to heart failure or death 7.

There is an urgent demand to develop novel treatments of severe cardiomyopathy and MI. Pharmacological discoveries, transplantation of cells into the MI area, and xenogeneic heart transplantation from a pig to a patient with heart failure were applied, nevertheless these approaches currently do not meet the clinical demand 8-10. Pharmacological interventions can slow down heart failure progression; yet they are not able to regenerate the diseased organ. Transplantation of various types of cells did not fully match the expectations yet and transplantation of a whole heart from a genetically modified pig failed after 7 weeks 11. Further research is necessary to bring the novel therapies from bench to bedside.

The discovery of human induced pluripotent stem cells (hiPSCs) in 2007 has led to a breakthrough in the field 12. The novel technology solves ethical issues, reduces the cost of research and allows to derive human CMs in standardized fashion *in vitro* 13, 14. As hiPSC differentiation technology rapidly evolves, first-generation protocols designed to generate only one specific cell type at a time are being complemented by more sophisticated methods to generate cardiac microtissues (cMTs) or organoids. Importantly, multicellular cardiac organoids (COs) are advancing the field of personalized medicine 15. COs are useful to study specific physiological pathways during stages of embryogenesis, to generate cardiac disease models, in pharmaceutical drug development, in 3D bio-printing, and for cardiac regeneration.

These 3D cMTs and/or COs can be applied for drug validations, although some hurdles such as high hands-on time for their production remain 16. Furthermore, an optimal and defined matrix for 3D gel culture of organoids is needed, and the limited scalability of the process and the lack of long-term culturing perspectives restricts the applicability of COs 17, 18. Of note, the current approaches of COs formation were developed to generate cMTs and/or COs for drug testing, but not as a platform to produce transplantable material for cell therapy. We put the emphasis on the development of an industrial scalable production with non-cryogenic transportation method of COs for pharmaceutical drug modelling and initiating cardiac cell therapy.

To address this, we developed a bioreactor-based, scalable protocol for generating transplantable COs containing CMs, ECs, CFs, and additional cardiac cell types. In prior work, we demonstrated >90% efficiency in differentiating hiPSCs to CMs in both 2D and 3D bioreactor formats, and >90% EC generation using serum-free conditions with VEGF, bFGF, PKA, and melatonin stimulation 13, 14, 19.

In this study, we integrated these protocols to create a unified differentiation strategy for generating COs either in 96-well plates for *in vitro* assays or scalable bioreactor culture for cell therapy following MI. We utilized a non-cryogenic international transportation approach, and we validated this approach using a translational porcine model of acute MI, assessing both its safety and preliminary efficacy. This strategy is a significant step towards the clinical translation of engineered cardiac tissues.

## Results

### Small scale production of COs from hiPSCs using 96-well plates

**Figure 1:**
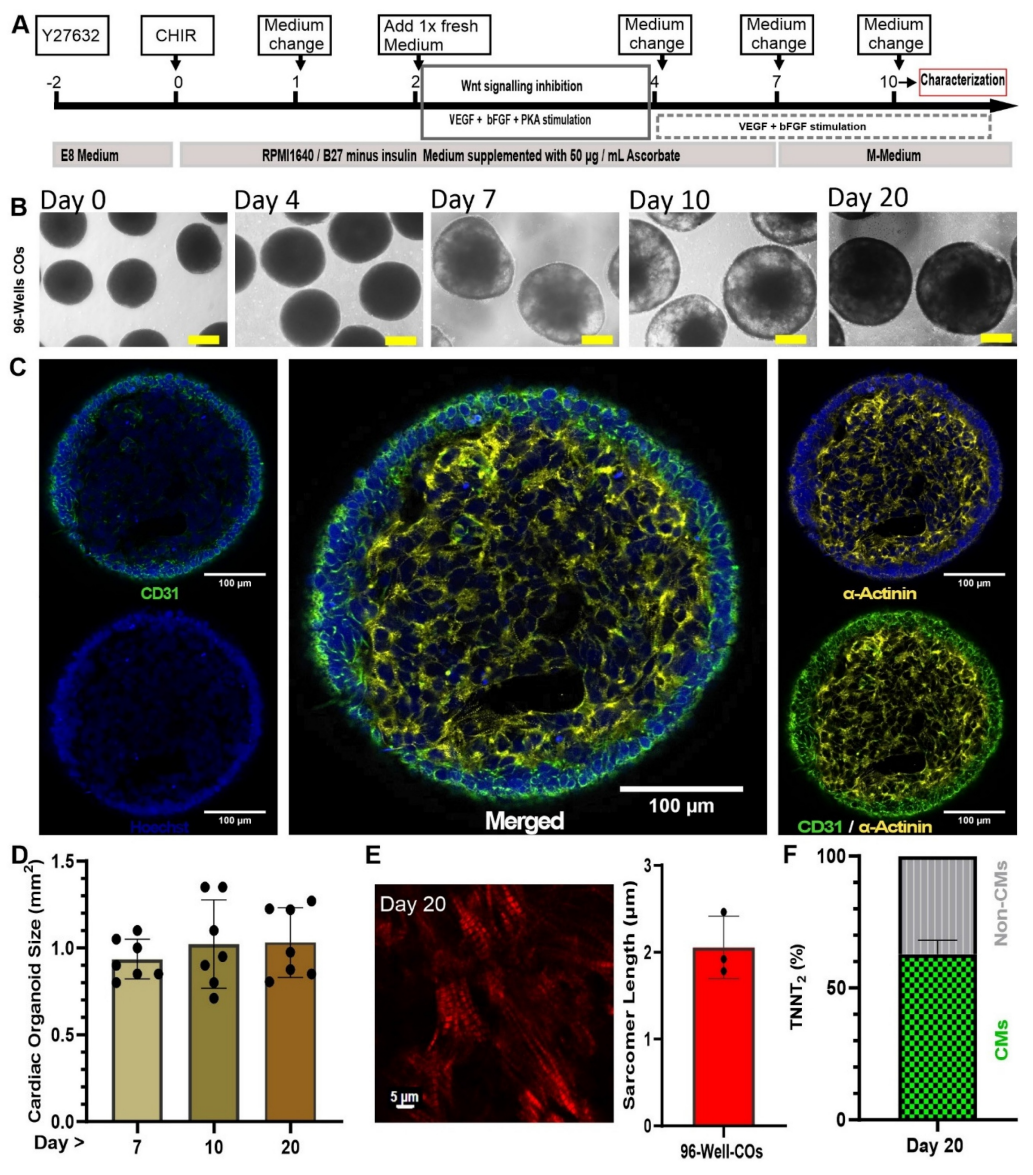
** Generation of small-scale COs inside 96-well plates.** (**A**) Optimized schematic process of CO differentiation. Scale bar: 250 µm (**B**) Transmission light imaging of COs cultured as a pool of multiple COs per dish on an orbital shaker. (**C**) Whole-mount CO immunostaining at day 10. The whole mount was scanned without embedding under a cover glass to preserve 3D structure. Cardiomyocytes are stained by antibodies against cardiac α-actinin (yellow) and endothelial cells by antibodies against CD31 (green). Nuclei stained by Hoechst (blue). (**D**) Growth kinetics of COs observed for 20 days. (**E**) Second harmonic generation imaging of sarcomeres in day 20 COs and determination of sarcomere length using multiphoton microscopy. Scale bar: 5 µm. (**F**) Analysis of flow cytometry of COs at day 20 with antibody against cardiomyocytes (cardiac troponin T, TNNT2) conjugated with FITC.

Human induced pluripotent stem cells formed compact spheroidal aggregates of hiPSCs after collection of the cells by centrifugal force in U-shape ultra-low attachment 96-well plates followed by 2 days of culture (**Figure 1A-B**). Induction of mesodermal progenitors via supplementing only CHIR99021 for 24 hours showed complete fate conversion (98-99%) as quantified by flow cytometry using anti-brachyury antibodies (data not shown) three days post-mesodermal induction. To find the optimal stage of mesoderm progenitors to develop into COs, microarray analysis was performed for non-induced cells (“None”) and days 1, 2, and 3 post-mesodermal induction. Microarray-transcriptomic and principal component analysis revealed that there was no difference between biological replications (N = 3) within groups, but there was distinctive separation between groups (**Figure S1A**).

Differential gene expression analysis showed differences between the mesoderm progenitors on day 1, 2 and 3 post induction, indicating a dynamic process of development (**Figure S1B**), where 715, 1315, and 750 differentially expressed genes of day 1 vs none, day 2 vs none, and day 3 vs none were identified (**Figure S1C**). To find out which biological pathways were active, we performed gene ontology for those genes that show a log 2 > 0.5 differential expression comparing day 1, 2 and 3 vs none (non-mesodermal induction; control) (**Table S1**)**.** The three ontology profiles revealed that Day 2 post-mesodermal induction was the most comprehensively in terms of capturing the multifaceted biological processes essential for coordinated cardiac and vascular differentiation. While all biological pathways encompassed fundamental mesodermal patterning and organogenesis pathways such as Wnt signalling and gastrulation, Day 2 uniquely integrated key processes underlying epithelial tube morphogenesis, branching morphogenesis, and cell junction assembly, which are critical for vascular network formation (**Table S2**). In contrast, Day 1, although rich in early mesodermal and organ developmental terms, lacked explicit emphasis on vascular morphogenesis and endothelial maturation. On Day 3, cardiac structural development and muscle differentiation pathways dominated but cell junction assembly, indispensable for vascular development, was underrepresented (**Table S2**). Importantly, day 2 also included transcripts related to nervous system development and neurovascular interactions, highlighting the interplay between neural and vascular elements during organogenesis (**Figure S1D, and Table S2**). Therefore, Day 2 post-mesodermal induction was the optimal time point to further differentiate mesodermal progenitors towards cardiac mesoderm using Wnt signalling inhibition (IWP2 and XAV939) and VEGF, bFGF and PKA stimulation (**Figure 1A**).

To further mature cardiac cells and support the prolonged maturation of COs, specifically from day 7 onwards (when COs usually started to contract, as shown in **Movie S1**), a maturation medium (“M-Medium”) was optimised in nine groups (n = 6 - 11 biological independent replications). COs cultivation in experimental media was performed from day 7 to day 10 followed by flow cytometry and statistical analysis of TNNT2 and VE-Cadherin-positive cells. Based on this analysis, CM (TNNT2) and EC (VE-Cadherin) fractions, respectively, showed non-significant (P > 0.05) difference between all nine groups (**Figure S2A-D**). Analysing trends, culture of COs from day 7 to 10 in M-Medium containing insulin, dexamethasone and fatty acids (palmic acid and oleic acid) showed top performance among nine experimental groups (e.g., lowest S.D. = 20.4 for VE-cadherin%, and highest actual confidence level = 98.83 for VE-Cadherin% and TNNT2% with highest sample size = 11) (**Figure S2E**) and was selected for further experiments. Optimized M-medium allowed us to culture COs with and without additional rT3 hormone supplementation (non-supplemented; control: n = 2, and rT3-supplemented: n = 5) from day 10 onwards. On day 35, COs electrophysiological properties were measured using sharp electrode recordings and showed that rT3 significantly increased action potential (AP) amplitude in comparison to non rT3 supplementation (91.71 ± 2.02 mV) vs non-treated (69.75 ± 13.93 mV), respectively, and rT3 produced a temporary depression (“notch”) after the initial upstroke of the AP indicative of functional K^+^i_to_ channels (**Figure S3A-B**). Additionally, the mean diastolic potential was lower in rT3-treated COs versus control (-71.60 ± 1.1 mV vs. -55.23 ± 9.11 mV, respectively), and the AP duration at 50% repolarization (APD50) (350 ± 90 ms vs. 517 ± 35 ms, respectively) and 90% repolarization (APD90) (557 ± 33 ms vs. 390 ± 90 ms, respectively) were prolonged and the maximal upstroke velocity was increased (13.90 ± 0.12 vs. 6.63 ± 4.83 V / s, respectively) (**Figure S3B**). Based on these findings, rT3 was supplemented for all further differentiation experiments.

On day 10, an immunofluorescence staining was performed and showed a core-shell structure with a core of CMs, positive for cardiac α-actinin, and an outer layer of ECs positive for CD31 (**Figure 1C**). The size of COs increased from day 7 to day 10 and remained constant between day 10 and day 20 (0.94 ± 0.12 mm, 1.023 ± 0.25 mm, and 1.031 ± 0.20 mm; respectively) (n = 7, technical replications) (**Figure 1D**).

Regarding the core-shell structure of the COs and the maturation status of the CMs, the distance between the myosin bands and the sarcomere length was calculated based on second harmonic imaging. This technique is capable of imaging myosin bundles without the need for staining. Sarcomere lengths were determined as 2.06 ± 0.36 µm (N = 3 biological replicates, with each data point representing n = 5 different spots analysed per CO) on day 20 of the CO differentiation process (**Figure 1E**). This distance is similar to that observed in mature cardiomyocytes 20.

To understand whether COs stay alive in prolonged culture without embedding in a gel matrix such as Matrigel, COs were continuously cultured in M-Medium till day 100. Immunofluorescence analysis of day 100 COs identified CMs positive for cardiac α-actinin marker at the core of COs. Sarcomere organization was still visible as indicated by a striated pattern of cardiac α-actinin. Non-myocytes had substantially proliferated resulting in a continuous growth of the COs (**Figure S4**). TUNEL assay confirmed that the fraction of apoptotic cells at day 100 and 213 were 4.6% and 13.8%, respectively. In comparison, the positive and negative controls contained 100% and 0.012% stained cells, respectively (**Figure S5**).

Lastly, on day 20 of COs differentiation, we did flow cytometry (FC) to determine the fractions (%) of TNNT2 positive cells and non-myocytes (Non-CMs). By FC it was determined that COs contained 62.8% ± 13 (n = 6) TNNT2 positive cells and 37.2% (n = 6) Non-CMs (**Figure 1F**).

### COs cellular composition and developmental status

**Figure 2:**
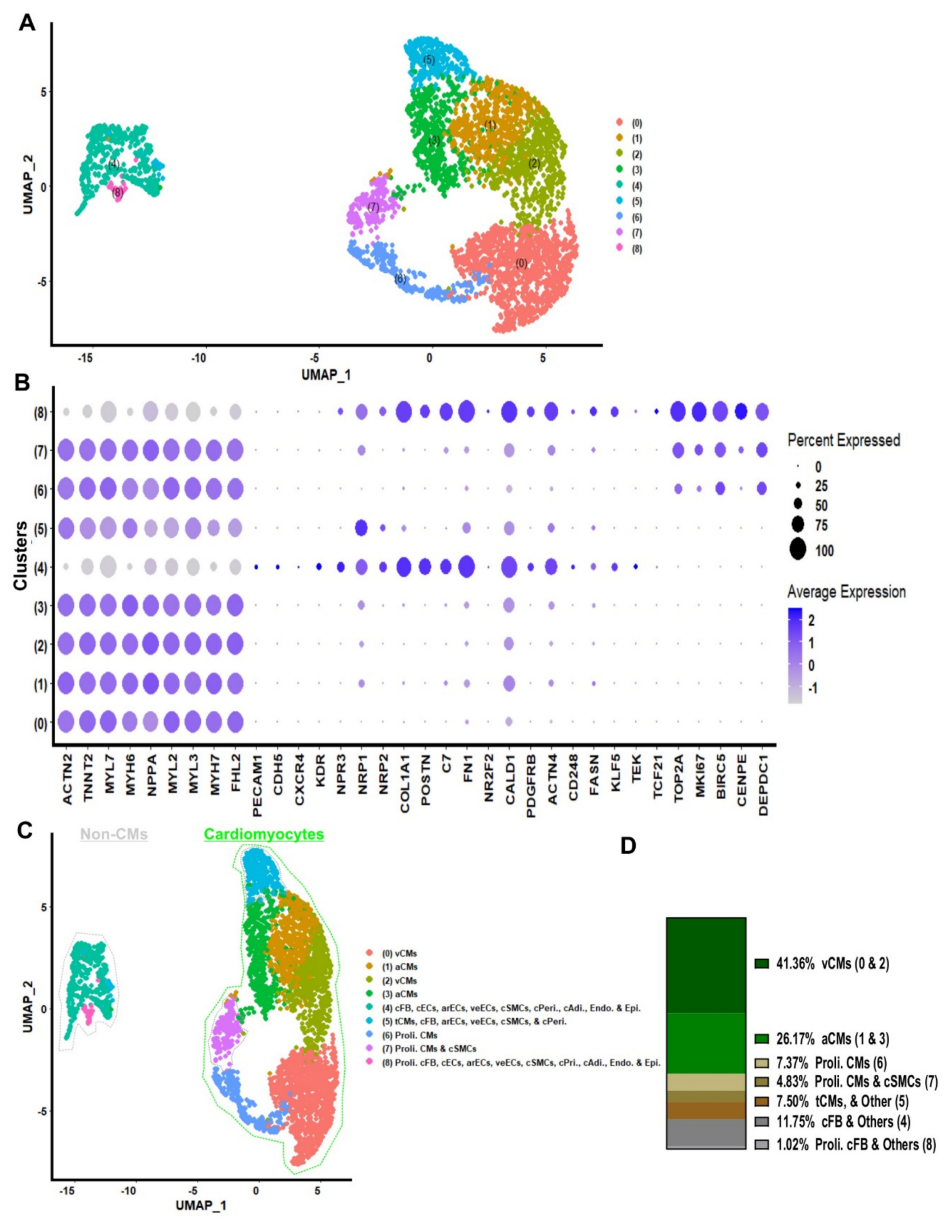
** single cell RNA sequencing of 96-well plate COs on day 20.** (**A**) scRNA-Seq data on day 20 of 96-well plate COs revealed 9 distinct clusters, Wilcoxon test was applied for differentially expressed markers (genes) per cluster, and these genes were compared with PanglaoDB^21^ and the Human Protein Atlas^22^ data bases, and with published article to annotated the cluster identity. (**B**) Dotplot was used to compare expression of marker transcripts in different clusters of 96-well plate COs. (**C**) Clusters annotation. (**D**) Percentage of cell clusters were analysed: ventricular-like CMs (v-CMs = 41.36%), atrial-like CMs (a-CMs = 26.17%), proliferative cardiomyocytes (prolif. CM = 7.37%), cardiac fibroblast and other cardiac cell types (cFB & Others = 11.75%), proliferative cardiomyocytes and cardiac smooth muscles (prolif. CMs & cSMCs = 4.83%), transitional cardiomyocytes and other cardiac cells types (tCMs & Others = 7.50%) and proliferative cardiac fibroblast and other cardiac cells (prolif. cFB & Others = 1.02%).

Ten to twenty COs were dissociated to single cells and non-integrated scRNA-seq analysis of day 20 COs (generated in 96-well plates) was performed (**Figure 2A**). Cluster identities were annotated by comparing the top significantly (P < 0.05) differentially expressed marker genes for each cluster (**Figure 2A and Table S3**) using the Wilcoxon statistical test to established databases, such as PanglaoDB and the Human Protein Atlas 21, 22. Cell type annotations were further confirmed on the ground of published cell markers (**Figure 2B, and Table S4**) 18, 23-25. COs at day 20 contained 41.36% ventricular cardiomyocytes, 26.17% atrial cardiomyocytes, 7.37% proliferative cardiomyocytes, 4.83% proliferative cardiomyocytes or cardiac smooth muscle cells, and 7.50% cells in transition, including cardiomyocytes and others such as cardiac fibroblasts and endothelial cells. The remaining 12.77% consisted of non-proliferative cells, including fibroblasts, endothelial cells, smooth muscle cells, pericytes, adipocytes, endocardium, and epicardium (**Figure 2C - D**). Interestingly, we did not detect a single remaining pluripotent cell in COs at day 20 by using scRNA-seq analysis (**Figure S4**). Additionally, the gene expression on single cell level indicated the co-expression of cardiac Troponin T (TNNT2) together with the ventricular marker genes regulatory light chain 2 and 3 (MYL2, MYL3) and myosin heavy chain alpha isoform (MYH6), as well as the mature form of Troponin I (TNNI3) was identified in a majority of cells which were allocated in the cardiomyocyte area (**Figure 2B, and Figure S7**). These results demonstrated that by day 20, the COs contained only cardiac cell types: CMs at relatively mature, transitional, and proliferative stages; cFBs; cECs; cSMCs; and other non-myocytes, both proliferative and non-proliferative (**Figure 2C - D**).

Furthermore, to gain more insights into the cellular composition of iPSC-COs at day 10 and 20 of differentiation, we performed integrated scRNAseq analysis (**Figure S8A**). Cluster identities were annotated by comparing the top positive differentially marker genes (**Table S5**) for each cluster to established databases in comparison with PanglaoDB and the Human Protein Atlasdatabases (**Figure S8A - B**), and cell type annotation further confirmed by published cardiac cell markers (**Figure S8B**) 21, 22. COs contained 32.5% ventricular cardiomyocytes, 24.1% atrial cardiomyocytes, and 8,4% proliferative cardiomyocytes. The remaining cells in COs comprised fibroblasts (13%), non-proliferative endothelial cells and pericytes (6.7%), and proliferative cells including fibroblast, endothelial, and smooth muscle cells (**Figure S8C - D**). Integrated scRNA-seq analysis further confirmed that cardiac organoids (COs) at both day 10 and day 20 differentiation time points contained only cardiac cell types.

Lastly, to determine the biological active pathways of COs, we performed a gene ontology analysis for differentially expressed genes (avg_log2FC > 0.5) among clusters of integrated scRNAseq data of COs day 10 and 20 (**Table S5**). The ontology analysis and selected top 33 positive active pathways revealed that active ontology pathways of COs were heart morphogenesis, striated muscle tissue development, cardiac tissue muscle development, striated muscle cell differentiation, muscle organ development, epithelial cell migration, mesenchymal development, cardiac chamber development, and cardiac chamber morphogenesis (**Figure S9A**). Moreover, we did gene expression level analysis for integrated scRNA-seq of day 10 and 20 COs, indicating that COs expressed high levels of NPPB, PRKACA and CORIN maturation markers, as well as RYR2, PLN and CAMK2B (**Figure S9B**). A collective analysis of the cardiac organoids revealed that they exclusively comprised cardiac lineage cell types at diverse developmental stages: relatively mature, transitional, and proliferative. No residual pluripotent cells were detected. These organoids continued to progress through cardiac development, exhibiting expression of key cardiomyocyte maturation markers, including NPPB, PRKACA, CORIN, PLN, and RYR2.

### Connexin 43 gap junctions and cardiomyocyte ultrastructural in COs

**Figure 3:**
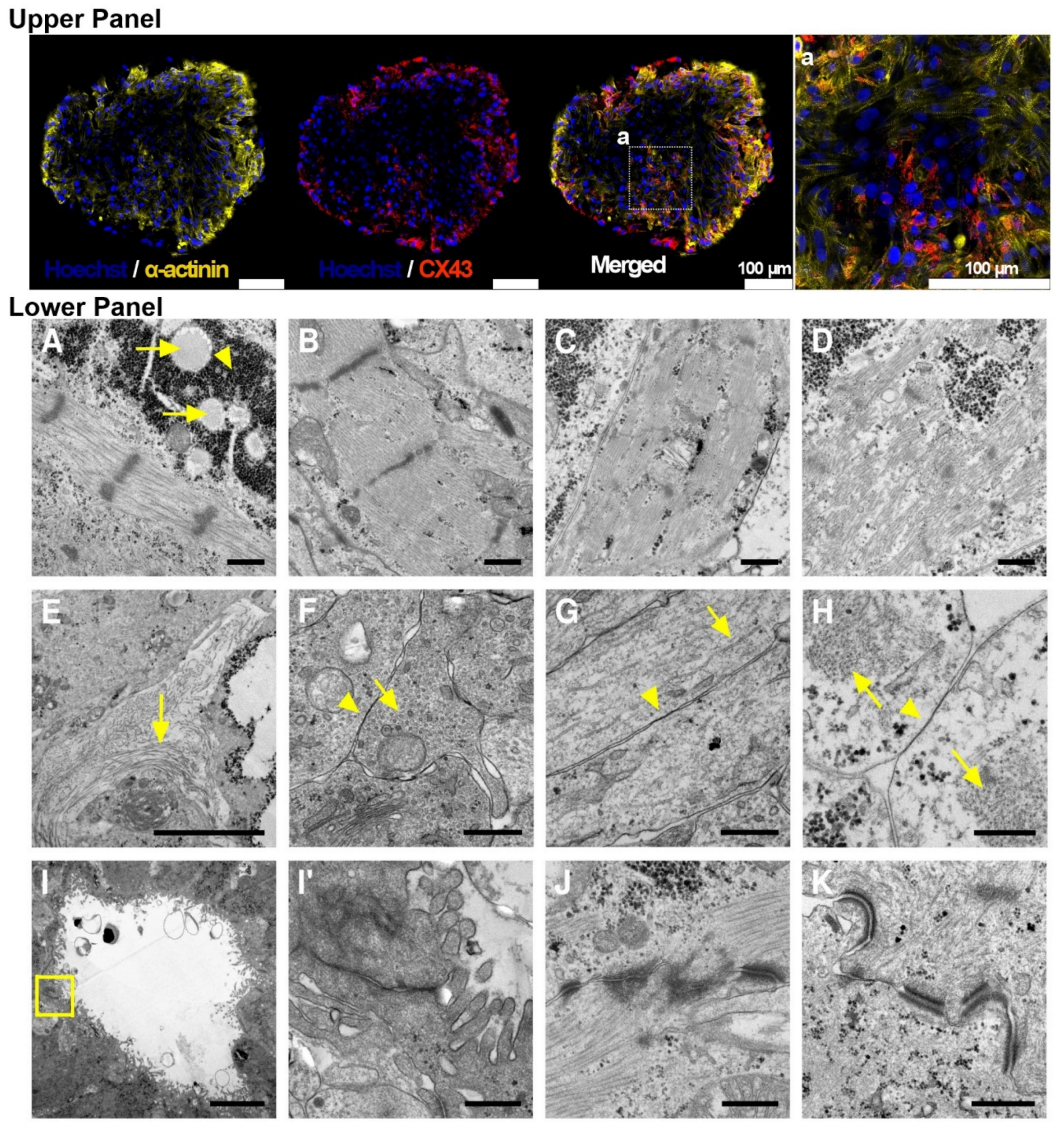
** Connexin 43 expression and ultra-structure of COs. Upper panel:** Whole-mount cardiac organoid immunostaining at day 30. The whole mount was scanned without embedding under a cover glass to preserve 3D structure. Cardiomyocytes are stained by antibodies against cardiac α-actinin (yellow) and connexin 43 (red). Nuclei stained by Hoechst (blue). Scale bar: 100 µm. **Lower panel:** Transmission electron microscopy of COs. (**A**) CMs (day 20) show myofibrils from which some are organized into sarcomere like structures, high amounts of glycogen (arrowheads) and lipid droplets (arrows) are found (**B**) CM (day 19) with sarcomeres and mitochondria (**C**) CM (day 40) with organized myofibrils (**D**) CM (day 40) with less organized myofibrils (**E**) Collagen can be found between non CMs (**F**) Numerous potential gap junctions (arrowhead) can be found between non CMs cells (day 30) which contain many microtubules (arrow) in crossection (**G**) longitudinal section of microtubule (arrow) and gap junction (arrowhead) containing cells. (**H**) potential gap junctions (arrowhead) between CMs with myofibrils (arrow), (**I**) Overview of a cavity with microvilli found in 30 days old CO, (**I´**) Enlargement of boxed area with potentially tight junctions between cells. (**J**) CMs (day 19) connected by an intercalated disc-like structure with organized myofibrils (**K**) and CMs (day 20) with less organized myofibrils.

To further confirm whether CMs in COs expressed Connexin 43 (CX43) gap junctions, and to assess the ultrastructure of cardiac cells, specifically CMs, at different CO differentiation time points, we performed whole-mount immunostaining on COs at day 30. The stained COs showed positive CX43 staining (red) within the core structure of the organoids, which also stained positive for cardiac α-actinin (**Figure 3**). The presence of CX43 at day 30 raised a question regarding the ultrastructural features before and after this time point. Therefore, we performed TEM analysis from day 19 to day 40.

At day 20, COs exhibited large energy stores in the form of glycogen and lipid droplets (**Figure 3**). A high degree of organization of contractile proteins into sarcomeres was identified as early as day 19 (**Figure 3**). At day 40, CMs within COs showed both well-organized sarcomeres and some with a lower degree of sarcomere organization (**Figure 3**). In the extracellular space, collagen deposits were observed (**Figure 3**). TEM also revealed the presence of non-myocyte cells rich in microtubules and coupled via potential gap junctions (**Figure 3**). Potential gap junctions were also found between myofibril-containing CMs (**Figure 3**). Cavities containing microvilli-bearing cells were observed (**Figure 3**), showing potential tight junctions between cells. Potential intercalated discs between CMs were also detected in day 20 COs (**Figure 3**).

Both whole-mount immunostaining and TEM not only further confirmed the cardiac cell composition of COs as revealed by scRNA-seq, but also demonstrated that COs expressed CX43 gap junctions. In addition, TEM revealed ultrastructural features that are consistent with both relatively mature and transitional cardiac cells, including CMs and non-myocytes. CMs exhibited a high degree of contractile sarcomere organization, and intercalated discs were observed between CMs from day 19 onwards.

### Cardiac organoid electrical activity

**Figure 4:**
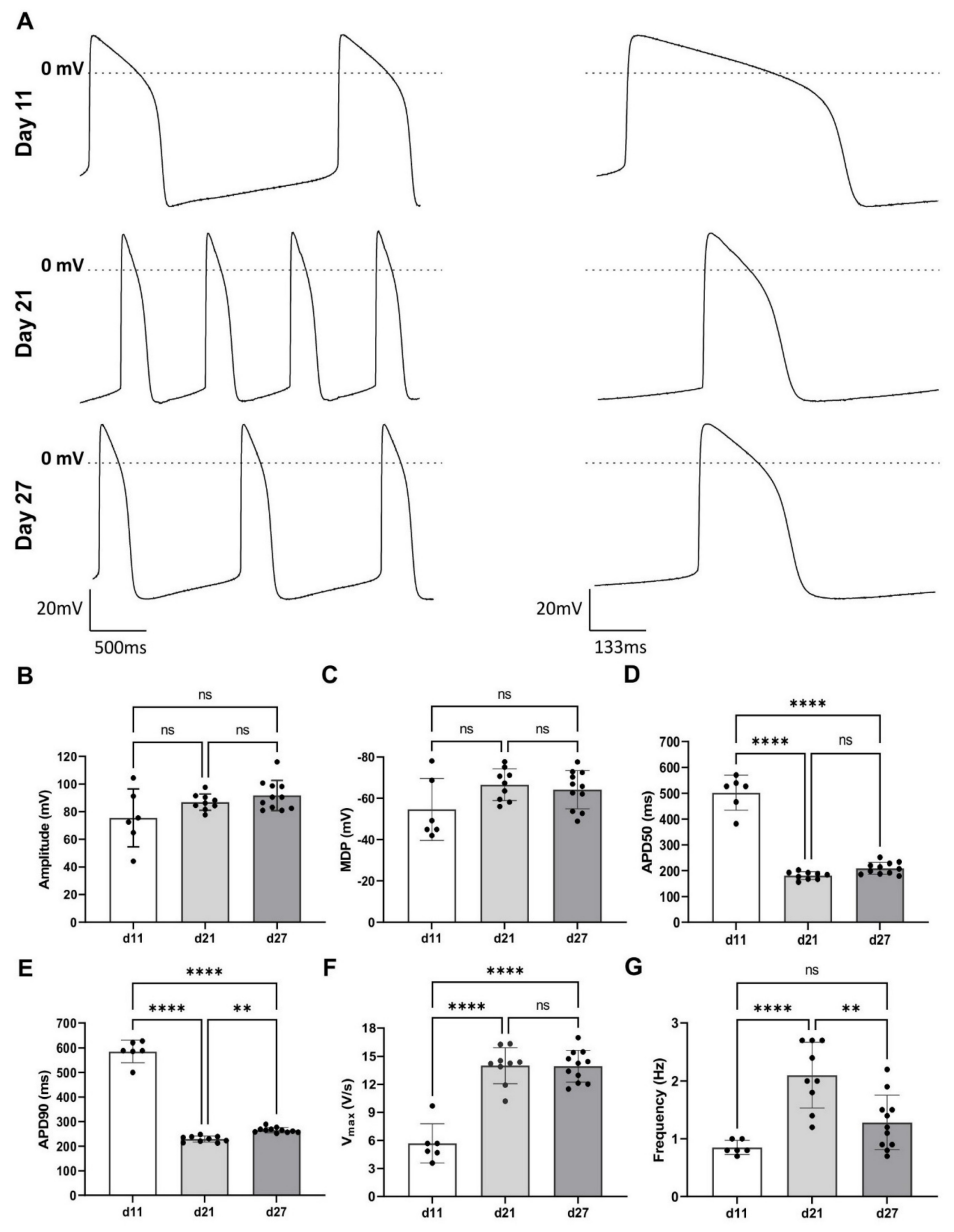
** Electrophysiology of COs by sharp electrode measurements.** (**A**) Action potential recordings at day 11, 21, and 27 (**B**) were analysed for amplitude, (**C**) mean diastolic potential MDP, (**D**) action potential duration APD50, (**E**) APD90, (**F**) maximum upstroke velocity, and (**G**) frequency of spontaneous contractions. *: p-value <0.01, **: p-value <0.001.

Our previous experimental data indicated that COs from day 19 onward represent the optimal time window in terms of cardiac cellular composition and maturation. To better understand the electrical activity of CMs within COs across early to late differentiation stages, we cultured COs in M-Medium from day 11 to day 27 and measured the APs of CMs within the COs using sharp electrode recordings. AP measurements were performed in the same COs over time to monitor the progression of electrical activity during long-term culture (**Figure 4A**).

Differences in AP properties were observed between day 11, day 21, and day 27. The AP amplitude increased from day 11 (75.46 ± 8.55 mV) to the day 21 (86.93 ± 1.96 mV) and day 27 (91.72 ± 3.28 mV; **Figure 4A - B**). The maximum diastolic potential (MDP) became more negative over time, shifting from -54.65 ± 6.13 mV (day 11) to -66.61 ± 2.56 mV (day 21) and -64.21 ± 2.79 mV (day 27), though these changes were not statistically significant (**Figure 4C**).

The APD50 and APD90 decreased significantly: from 501.07 ± 27.71 ms and 585.07 ± 118.47 ms at the day 11 to 180.60 ± 5.03 ms and 229.06 ± 4.09 ms at the day 21, and 204.56 ± 6.93 ms and 265.40 ± 3.2 ms at the day 27, respectively (**Figure 4D - E**). Upstroke velocity increased significantly from day 11 (5.7 ± 8.6 V / s) to day 21 (14.0 ± 0.64 V / s), with no further increase at day 27 (13.94 ± 0.51 V/s) (**Figure 4F**). The spontaneous contraction frequency rose significantly from 0.8 ± 0.1 Hz (day 11) to 2.1 ± 0.2 Hz (day 21), followed by a decrease to 1.3 ± 0.1 Hz (day 27), though these changes were not statistically significant (**Figure 4G**).

To support the sharp electrode findings, specifically that COs from day 19 onward display matured electrical activity, we also performed optical mapping of APs on day 21 in the COs derived from NP0040-DSR 14 and NP0040 (wild-type) hiPSC lines (**Figure S10**). In the NP0040-DSR line, 6 out of 9 COs were spontaneously active (**Figure S10A**), with an average beat rate of 157 ± 3.4 beats per minute and an AP duration of 146.3 ± 1.7 ms. In the NP0040 (wild-type) batch, only one CO showed spontaneous activity, exhibiting a single AP with a duration of 242 ms (n = 1) (**Figure S10B - C**). None of the COs responded to bipolar stimulation. COs had an approximate diameter of 2 mm, and activation was completed within ~20 ms, corresponding to a conduction velocity of >10 cm/s (**Figure S10D - E**).

Together, sharp electrode and optical mapping data demonstrate that hiPSC-derived COs undergo progressive electrophysiological maturation between day 11 and day 27, with significant improvements in AP amplitude, upstroke velocity, and shortening of AP duration. Optical mapping confirmed rapid tissue-level conduction and more robust spontaneous activity by day 21, particularly in the NP0040-DSR line. These findings validate the use of COs from day 19 onward as a reliable platform for modelling mature cardiac electrophysiology and potential disease-specific electrical phenotypes, and/or for scalable industrial COs productions for regeneration cardiac cell therapy.

### Large scale of COs from hiPSCs production using suspension bioreactor culture

**Figure 5:**
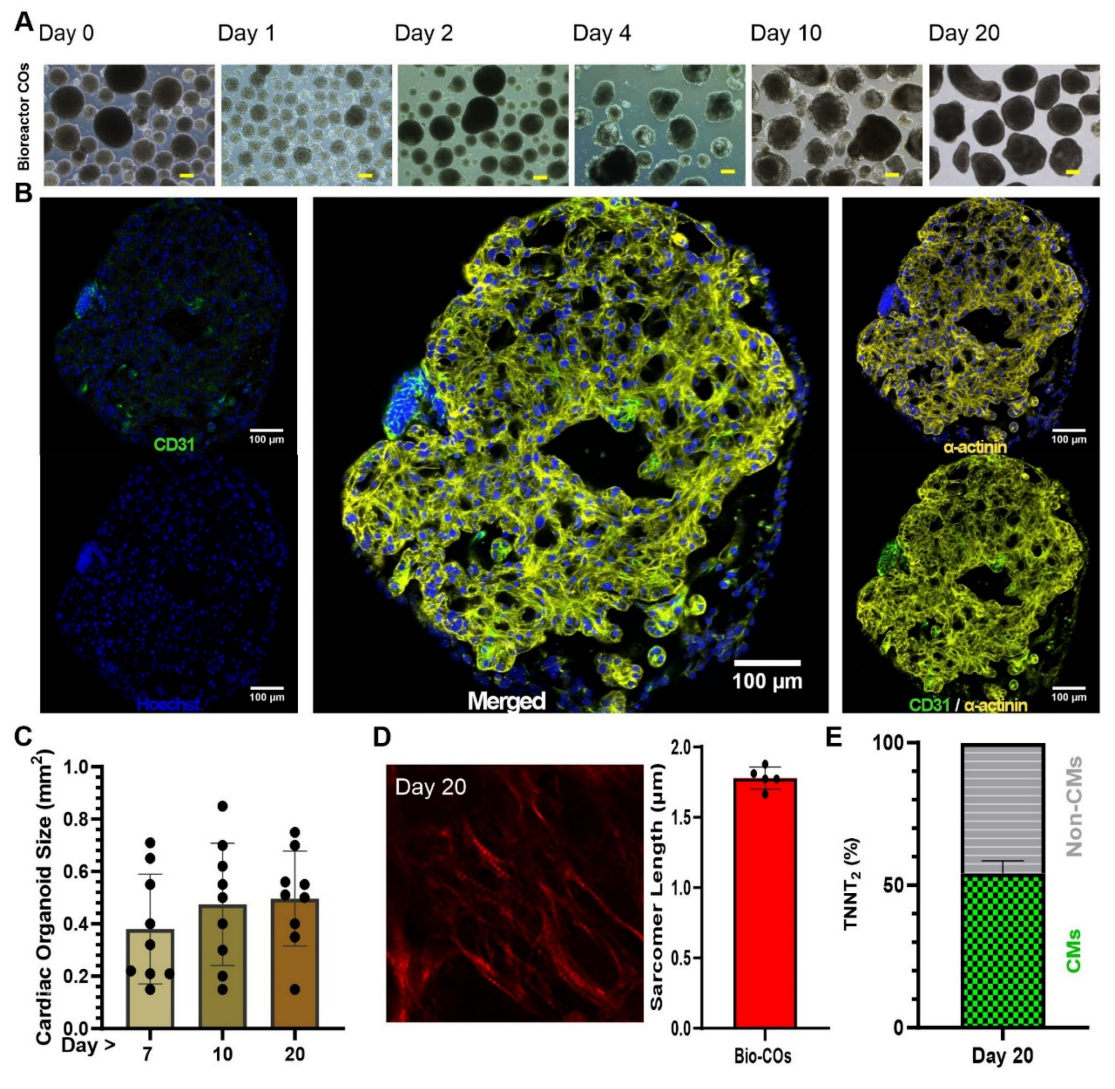
** Generation of COs in bioreactor suspension culture.** (**A**) Morphology of COs in suspension at different days of culture. Scale bar: 250µm (**B**) Whole mount immunofluorescence staining of COs for cardiac α-actinin (yellow) and CD31 (green) on day 10. Nuclei stained by Hoechst (blue). (**C**) Size distribution of COs. (**D**) Second harmonic generation imaging and sarcomere length of COs day 20. (**E**) Quantification of flow cytometry on day 20.

The generation of COs in 96-well plates has been demonstrated to yield consistent results. However, its application in the production of large quantities of CO for regenerative therapies is constrained by inherent limitations. Therefore, we applied the same 96-well COs method in a suspension bioreactor culture system, and a number of 7000 COs per 100 mL were generated after an initial inoculation of the bioreactor with a single cell suspension of 30 x 10^6^ hiPSCs and continued culture of COs for 20 days. A key step in the suspension bioreactor protocol was adjusting EB density to 70 EBs / mL at day 0 of COs differentiation; apart from this no further changes were introduced to the established protocol. When compared to COs generated in individual wells of 96-well plates, COs produced in stirred suspension bioreactor culture appeared to be more heterogeneous in size (**Figure 5A**). As with the 96-well plate, the COs started to contract from day 7 onwards (**Movie S2**).

Whole-mount immunofluorescence staining was performed on day 10 of COs differentiation. Confocal imaging of stained COs showed areas that stained positive for CD31 (green) and α-actinin (yellow), and revealed a trabecular structure of the COs at day 10 (**Figure 5B**). For confirmation and comparison between COs formed in 96-well plates and COs formed in bioreactor suspension culture, paraffin sections were generated and stained by haematoxylin and eosin staining (**Figure S11**). The histological staining confirms the trabecular nature of the myocardial tissue inside COs and suggests the presence of open spaces.

The COs exhibited an upward trend in size as indicated by measurements taken on days 7 (0.38 ± 0.2 mm), day 10 (0.48 ± 0.24 mm) and day 20 (0.5 ± 0.18 mm) of differentiation (**Figure 5C**). Data was collected from 9 replications in order to validate batch to batch consistency. We used second harmonic generation images to determine a sarcomere length, which was about 1.77 ± 0.07 µm (n = 5; different areas of the same organoid) (**Figure 5D**).

In order to determine CMs fraction, COs were collected on day 20, dissociated to single cells and stained with antibodies against TNNT2. Flow cytometry showed 54.17 ± 4.35% of TNNT2 positive cells (CMs) at day 20 (n = 9), and the remaining cells were Non-CMs (**Figure 5E**).

**Figure 6:**
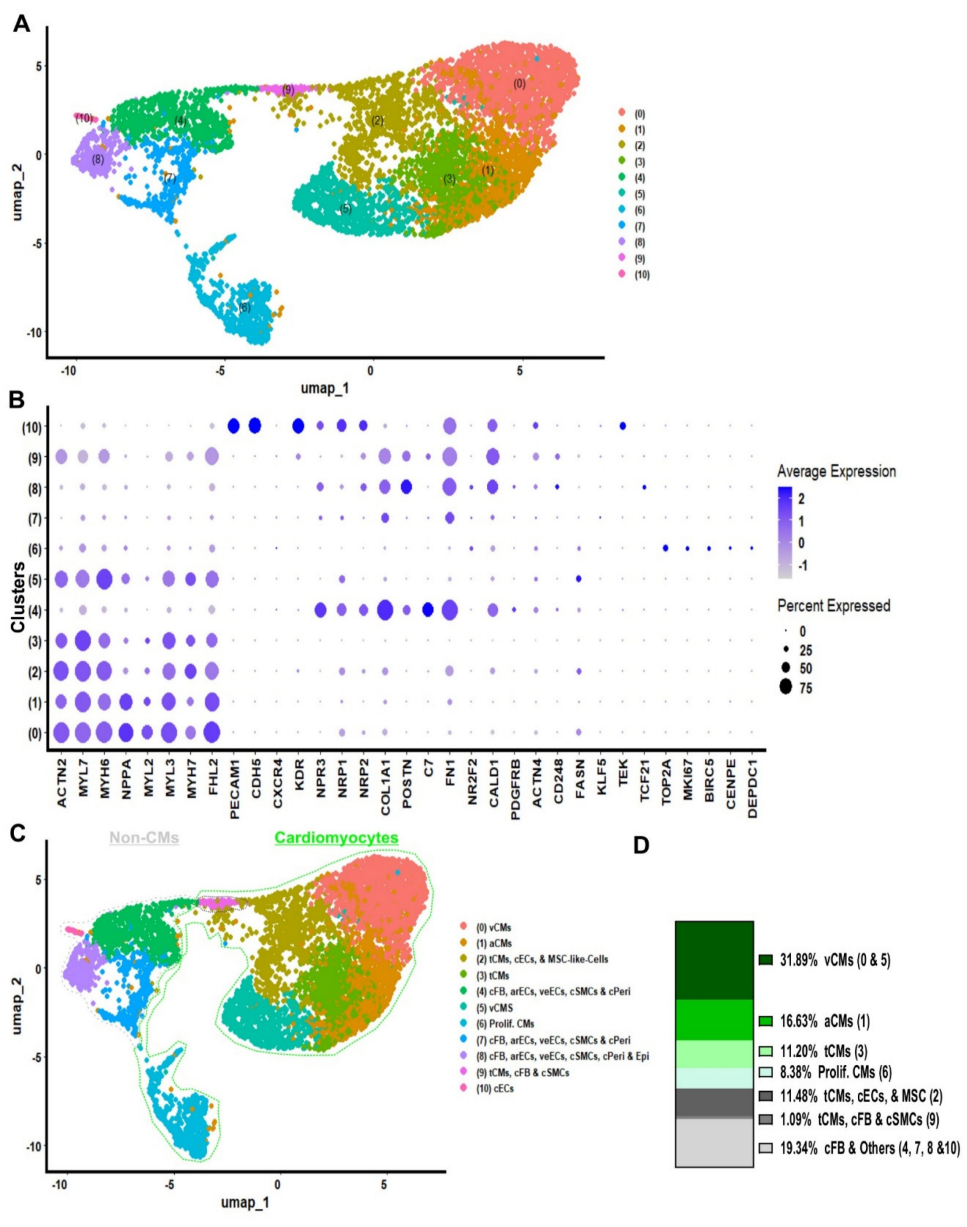
** Single-cell RNA sequencing of bioreactors COs on day 20**. (**A**) sc-RNA-Seq data on 20 of bioreactor COs was processed, normalized, scaled, KNN applied to cluster integrated anchored data, UMAP plot revealed 11 distinct clusters, Wilcoxon test was applied for differentially expressed markers (genes) per cluster, and these genes were compared with PanglaoDB and the Human Protein Atlas data bases, and with published article to annotate the cluster identity. (**B**) Dotplot was used to illustrate expression of marker transcripts in bioreactor COs at day 20. (**C**) Clusters annotation. (**D**) Quantification of annotated clusters: ventricular-like CMs (v-CMs = 31.89%), atrial-like CMs (a-CMs = 16.63%), proliferative cardiomyocytes (prolif CM = 8.38%), cardiac fibroblast and other cardiac cell types (cFB ; CFs & Others = 11.75%), transition cardiomyocytes (tCMs = 11.20), proliferative cardiomyocytes, cardiac endothelial cells and mesenchymal like stem cells (tCMs, cECs, & MSC = 11.48%), transition cardiomyocytes, cardiac fibroblast and smooth muscle cells (tCMs, cFB, cSMCs = 1.09%) and cardiac fibroblast and other cardiac cells (cFB & Others = 19.34%).

To resolve CM subtypes and characterize the cellular identity of the non-CM fraction along with their developmental status, we performed scRNA-seq on bioreactor derived COs at day 20 COs (**Figure 6A**). Cluster identities were annotated using the same comparative method applied to COs derived from 96-well plates. Significantly upregulated genes were identified in each cluster (Wilcoxon rank-sum test) and cross-referenced with PanglaoDB and the Human Protein Atlas databases (**Table S6**). Cell type annotations were further validated using established cardiac cell markers from the literature (**Figure 6B and Table S7**).

Day 20 COs from the bioreactor cultures comprised distinct cardiac cell populations, including ventricular CMs (31.89%), atrial CMs (16.63%), transitional CMs co-expressing ventricular and atrial markers (11.20%), proliferative CMs (8.38%), other cardiac-resident cell types such as transitional CMs, cardiac ECs, stromal-like mesenchymal cells, cardiac SMCs, and CFs (12.57%), and additional populations included cardiac fibroblast, vascular endothelial cells, smooth muscle cells, pericytes, and epicardial cells (19.34%) (**Figure 6C - D**). These findings demonstrate that COs generated in bioreactor suspension cultures at day 20 predominantly contain cardiac-specific cell types, similar to those observed in 96-well-derived COs.

To validate the fidelity of our hiPSC- COs generated using 96-well plates on orbital shakers and stirred-tank bioreactor suspension cultures, we performed an integrated scRNA-seq analysis. This included datasets from 96-well plate-derived COs at day 10 (96Well-Day 10) and day 20 (96Well-Day 20), bioreactor-derived COs at day 20 (Biorea.-Day 20), and a previously published scRNA-seq dataset of the human fetal heart at gestational week 6 (HHW6) (**Figure S12**) 26.

Uniform manifold approximation and projection analysis of the integrated dataset identified 18 distinct cellular clusters. The overall transcriptomic landscape revealed notable similarities between the hiPSC-derived COs and HHW6. However, slight differences in cellular composition were observed between COs derived from 96-well formats and those generated in bioreactor cultures (**Figure S12A**).

To gain insight of cluster identity, clusters were annotated by comparison with published cardiac markers. Bioreactor COs at day 20 showed an increased proportion of cells in cluster 10 - proliferative cardiac cells, whereas the relative abundance of this cluster declined from day 10 to day 20 in 96-well-derived COs. Cluster 3 (transitional CMs) was also enriched in bioreactor-derived COs, while cluster 9 (proliferative CMs) was more prominent in shaker-incubated COs (**Figure S12A - B**).

Interestingly, cluster 17, annotated as myeloid cells based on specific marker gene expression, was exclusively present in the bioreactor COs (only few cells) and HHW6, but absent in the 96-well-derived COs (**Figure S12A**). The remaining clusters represented a range of cardiac lineages, including ventricular and atrial CMs, ECs, SMCs, pericytes, and fibroblasts. The endocardial marker TEK was expressed in a subset of ECs (cluster 14), while the epicardial marker TCF21 was detected in a subpopulation of cluster 5, which predominantly co-expressed fibroblast and SMC markers. Expression of NRP1 and NRP2, markers associated with vascular endothelium, was also enriched in cluster 14. Notably, cluster 9 comprised CMs co-expressing proliferative markers such as TOP2A, MKI67, and DEPDC1, the latter being implicated in mitotic progression (**Figure S12A - B**). Together, these results demonstrate that the transcriptomic profiles of hiPSC-derived COs closely recapitulate key cellular features of the human fetal heart at week 6 of gestation, supporting their utility as a model for early human cardiac development.

Finally, to further contextualize our findings, we performed an integrated analysis incorporating publicly available scRNAseq datasets from CO and epicardioid differentiation protocols reported by Volmert *et al*. (Volmert-EMM2), Meier *et al*. (Meie-Day30), and Silva *et al*. (Silva-Day 100), and compared them to our 96-well-plate at day 133 COs generated in 96-well plates (96Well-Day 133) 24, 27, 28. Across the integrated dataset, 21 distinct clusters were identified (**Figure S13A**).

Organoids derived from the protocols of Volmert *et al*. and Meier *et al*., both cultured for 30 days, exhibited highly similar cellular compositions. In contrast, COs from Silva *et al*., cultured for 100 days, showed a notably higher proportion of fibroblasts (cluster 1), as well as a distinct enrichment of cells in cluster 7, putatively annotated as SMCs.

Our 96-well plate COs at day 133 displayed CM-enriched clusters (clusters 3, 5, 6, 8, and 10), comparable to those observed in Silva *et al*.'s day-100 COs. However, a marked difference was the substantial expansion of fibroblast populations in our samples, localized to clusters 1, 2, and 4. This expansion was observed in COs cultured in suspension without embedding in an extracellular matrix, suggesting that the absence of a gel matrix may influence fibroblast proliferation and structural remodelling in long-term cultures. Interestingly, clusters 13 and 14, annotated as proliferative CMs, were predominantly detected in early-stage COs, specifically those from Volmert-EMM2 and Meier *et al*.-Day 30. However, these proliferative CM populations were also present, albeit to a lesser extent, in later-stage organoids from Silva *et al*. (Day 100) and in our day-133 COs (**Figure S13A - B**). This observation supports the notion that COs can sustain survive and proliferative CM populations over extended culture durations, underscoring their potential as a model system for investigating human cardiac development across developmental stages.

### Non-cryogenic COs oversea distribution

To evaluate the feasibility of transporting COs and employing them in pharmacological testing, we distributed 7,000 COs into two separate 15 mL centrifuge tubes, each containing 3,500 COs fully immersed in M-Medium. These were shipped from Cologne, Germany, to the IGTP laboratory in Barcelona, Spain, at differentiation days 9 - 24. After 48 hours of shipment without cryopreservation, COs were acclimatized in M-Medium under rocking shaking at 37°C for 24 - 48 hours. A representative CO was then placed on a high-density microelectrode array (HD-MEA) (**Figure S14A, Movie S3, S4**), and its activity was recorded and visualized as an activity map (**Figure S14B**). A second, 30-day-old CO was used for subsequent analyses (**Figure S14C - G**). This organoid displayed spontaneous and synchronized compound field potentials (CFPs), as demonstrated by voltage traces from three representative electrodes (**Figure S14C**), reflecting its intrinsic beating rhythm and providing a reference for pharmacological modulation.

The electrical behavior, synchronicity, and beating regularity were further illustrated in CFP raster plots (**Figure S14D**), confirming intrinsic electrical activity with a stable pacing rate of 54.09 ± 2.35 cfp / min under control conditions. Upon administration of 5 µM adrenaline, the beating rate increased to 96.46 ± 2.26 cfp / min (**Figure S14G**), consistent with β-adrenergic responsiveness. Adrenaline also enhanced CFP amplitude, indicating increased excitability: The S-peak minimum amplitude rose from 48.59 ± 0.15 µV to 73.43 ± 0.13 µV, while the R-peak maximum increased from 53.35 ± 0.10 µV to 71.29 ± 0.09 µV (**Figure S14E**). Propagation maps (**Figure S14F**) showed no notable change in signal directionality, suggesting that enhanced electrical activity did not compromise conduction coordination.

Following exposure to 0.1 mM potassium chloride (KCl), the beating rate remained elevated at 98.80 ± 1.77 cfp / min (**Figure S14G**). Excitability further increased, as reflected in the elevated S-peak amplitude of 82.27 ± 0.18 µV (**Figure S14E**), while the R-peak amplitude declined slightly to 61.26 ± 0.08 µV. This suggests a differential modulation of depolarization and repolarization. Importantly, while rhythmicity and synchrony were maintained, propagation maps (**Figure S14F**) revealed a reversal in conduction direction, indicating that KCl disrupted spatial coordination of electrical signals. Additionally, the coefficient of variation of CFP intervals rose from 0.70 ± 0.02 ms under control conditions to 0.81 ± 0.02 ms with adrenaline, and further to 1.02 ± 0.02 ms with KCl, suggesting progressive destabilization of beat-to-beat regularity (**Figure S14G**).

These findings demonstrate the feasibility of large-scale CO production, inter-laboratory shipment, and functional assessment using HD-MEA. Furthermore, COs exhibit pharmacologically responsive and dynamically regulated electrophysiological properties, making them a promising platform for drug screening and disease modeling.

### COs validation after acute MI

### Study population and MI induction

To evaluate the therapeutic potential of cardiac cell therapy, 3,500 COs were transplanted into a porcine model of acute MI. As previously noted, the dosage per pig was selected based on the bioreactor's total yield of ~7,000 COs per batch within a 250 mL vessel. For initial safety and feasibility evaluation, this batch was divided into two equal groups of 3,500 COs, allowing assessment of potential adverse effects and therapeutic outcomes before proceeding to future dose-ranging studies. This approach ensured rigorous safety profiling while generating meaningful functional data. Thirteen animals underwent MI induction and were allocated into three groups: Long-term Control (n = 4), Long-term CO (n = 7), and Short-term CO (n = 2). One animal (Long-term CO) died during surgery due to ventricular fibrillation, leaving 12 animals in the study. Another animal (Long-term CO) died at 29 days post-MI due to pulmonary complications from immunosuppression therapy, resulting in incomplete 30-day CMR and HDM data for this case. The final dataset included 12 animals: Long-term Control (n = 4), Long-term CO (n = 6, including 5 with complete CMR and HDM data), and Short-term CO (n = 2).

### Cardiac electrocardiographic monitoring

Holter monitoring was completed in 7 animals of Long-term follow-up group (Control, n = 4; CO, n = 3). All animals but one in control group, presented with NSVT and MSVT during the first 48 hours following surgery, with no relevant arrhythmic events thereafter in either group. PVCs were common in both groups within the first 48 hours, with significant decline afterwards. Increased automaticity was established as the VT mechanism (Figure S15), both sustained and non-sustained events. Postoperative atrial fibrillation occurred in 2 animals per group, with no episodes beyond 48 hours. Only 1 animal in the control group presented with short episodes of complete atrioventricular block (maximum pause < 2 seconds). Programmed ventricular stimulation did not induce VT in any animal (Table 1).

### CMR imaging

Although this study was primarily designed to evaluate the survival and engraftment of COs in the injured porcine heart, rather than to optimize assessment of global cardiac functional outcomes, late gadolinium enhancement cardiovascular magnetic resonance (LGE-CMR) was performed at 2 and 30 days post-MI in the long-term follow-up Control (n = 4) and CO-treated (n = 5) groups. As expected, cardiac function and infarct size were comparable between groups at 2 days post-MI (Table 2).

The distribution of *in vi*vo data was assessed by visual inspection of quantile - quantile (Q - Q) plots. Given the small sample size, Q-Q plots were preferred over formal normality tests (e.g., Shapiro-Wilk or Kolmogorov-Smirnov), which have limited power in small samples and may fail to detect meaningful deviations from normality. Visual inspection allows evaluation of distributional shape, symmetry, tail behavior, and potential outliers. Q - Q plots revealed no substantial departures from normality for any variable (Figure S16). Accordingly, parametric statistical methods were considered appropriate. Paired t-tests were used to compare measurements between 2 and 30 days within each long-term group.

**Figure 7:**
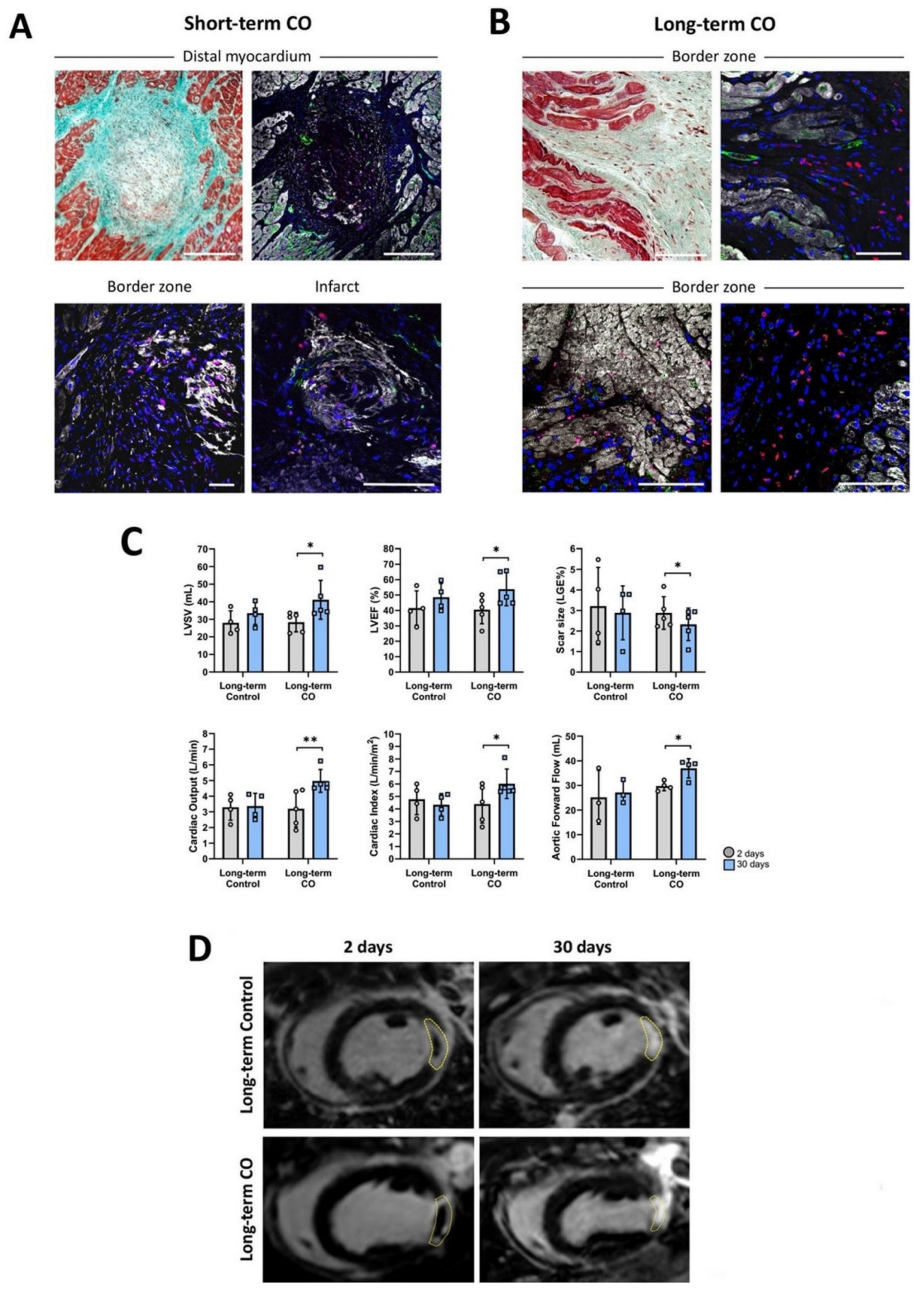
** CO transplantation in a model of acute MI in pigs.** (**A, Top**) Representative photographs of cardiac slices under Masson's trichrome staining and its respective image under immunohistochemistry against cardiac Troponin I (cTnI) (white), Human Nuclei Antigen (HNA) (red), CD31 (green) and nuclei (blue) showing the presence of human cells in healthy myocardium, and in the border and infarct zones infarct core (A, Bottom) in the Short-term CO group. (**B**) Images of border zone after Masson's trichrome (left) and its respective image under immunohistochemistry against cardiac cTnI (white), HNA (red), von Willebrand factor (vWF) (green) and nuclei (blue) showing the presence of human cells in a Long-term CO treated animal. Scale bars = 100μm. (**C**) Bar graphs displaying significant differences (*p < 0.05; **p < 0.01). in Long-term CO animals in terms of LVEF (0 = 0.034), Scar size (% of LGE Mass) (p = 0.029), Aortic Forward Flow (p = 0.040), Cardiac Output (p = 0.010), Cardiac Index (p = 0.048) and LVSV (p = 0.050) after paired T-test between 2 (grey) and 30 (blue) days of follow up. (**D**) Representative images of LGE-CMR of Long-term Control (up) and CO (down) animals after 2 (left) and 30 (right) days post-MI. Yellow dotted circles display the MI scar.

Over time, no significant differences were detected in the long-term Control group. In contrast, CO-treated pigs demonstrated significant improvements in left ventricular ejection fraction (LVEF; p = 0.034), scar size represented as the percentage of LGE mass (p = 0.029), aortic forward flow (p = 0.040), cardiac output (p = 0.010), and cardiac index (p = 0.048). Trends toward improvement were also observed for left ventricular stroke volume (LVSV; p = 0.050), extracellular volume (p = 0.085), indexed LV end-systolic volume (iLVESV; p = 0.056), and indexed RV end-diastolic volume (iRVEDV; p = 0.078), while left ventricular mass increased from 69.2 ± 4.1 g at 2 d post-MI to 75.9 ± 10.9 g at 30 d post-MI (p = 0.242). (Table 2; Figure 7C - D).

### Electroanatomic high-density mapping

Nine animals (Long-term Control, n = 4; Long-term, CO n = 5) underwent invasive endocardial LV HDM to assess the electrophysiological properties of the ischemic myocardium. Treated animals showed smaller low voltage (< 1.5 mV) areas compared to controls (0.5 ± 0.8 cm^2^ versus 1.3 ± 0.3 cm^2^, respectively), although this difference did not reach statistical significance. Consistent with infarct size, none of the animals displayed areas with voltages below 0.1 mV or deceleration zones (Table 3).

### Histological and immunohistofluorescence analysis

In all CO-treated animals (both 8 - days and 30 - days post-transplantation), immunohistofluorescence in frozen slices revealed the presence of HNA-positive cells (**Figure 7**), confirming the survival and engraftment of transplanted COs.

At 8 days post-transplantation, intact COs were identified within healthy myocardium, border zone, and in the core of the myocardial scar (**Figure 7A**). Within these organoids, two distinct HNA-positive cell populations were observed: 1) HNA^+^ CD31^+^ cells predominantly located at the peripheral margins of the clusters, and 2) HNA^+^ cTnI^+^ cells distributed throughout the organoids. By 30 days post-transplantation, intact spherical organoids were no longer detected. Instead, clusters of individual HNA^+^ cTnI^+^ and HNA^+^ vWF^+^ cells were observed, predominantly in the border zones and within the myocardial scar (**Figure 7B**). These findings suggest a progressive disaggregation of COs over time, accompanied by migration and integration of organoid-derived cells into the surrounding host tissue.

In parallel, myocardial scar ECM remodeling was evaluated using Picrosirius Red staining to quantify collagen composition at 30 days post-transplantation. Although differences did not reach statistical significance, CO-treated animals exhibited reduced collagen type I content, increased collagen type III content, and a lower collagen I : III ratio compared to control animals. These changes support a potential paracrine contribution of COs to scar remodeling.

### Spatial transcriptomics

**Figure 8:**
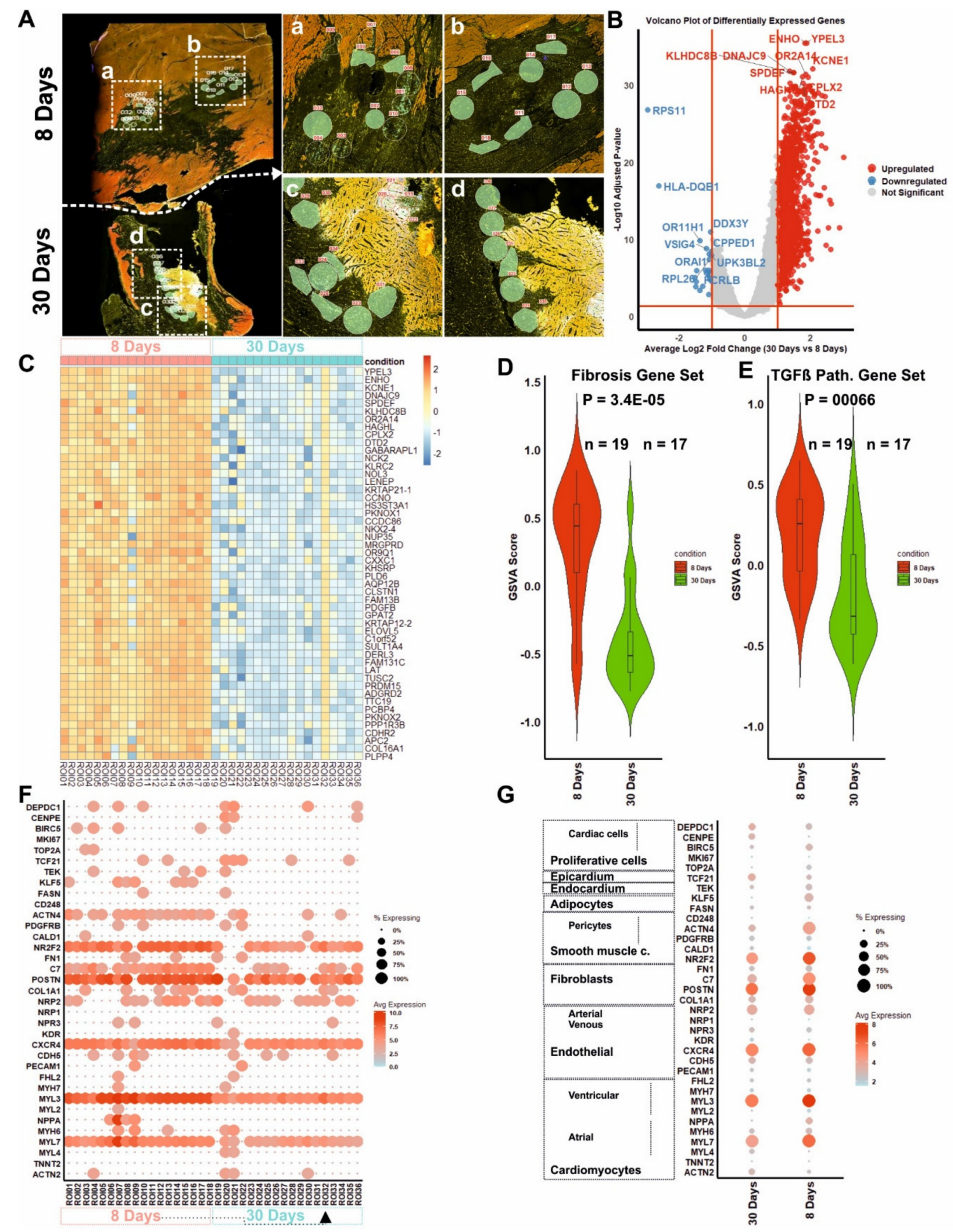
** GeoMx spatial transcriptomic profiling of porcine cardiac tissue at 8 and 30 days post-transplantation.** (**A**) Representative GeoMx digital spatial profiling images of OCT porcine cardiac tissue sections at 8 days (top) and 30 days (bottom) post-transplantation. Regions of interest (ROIs) were selected from human cell-enriched areas based on Human Nuclear Antigen marker detection. (**B**) Volcano plot showing differentially expressed genes between 30-day and 8-day ROIs. Upregulated (red) and downregulated (blue) genes are highlighted, where 30 days (n = 17 ROIs) vs 8 days (n = 19 ROIs); statistical significance was determined using adjusted P < 0.05. (**C**) Heat map of selected differentially expressed genes across all ROIs for top 50 positive differentially expressed genes. Each column represents an individual spatially resolved myocardial region, and each row represents a gene. (**D,E**) Gene set variation analysis (GSVA) scores of fibrosis-related (D) and TGF-β signaling pathway-related (E) gene sets, showing significantly decreased activity at 30 days (Wilcoxon rank-sum test, P < 0.05). (**F**) Dot plot showing the expression of representative human cardiac cell markers in ROIs at 8 (included ROI32) and 30 days. Dot size indicates the percentage of ROIs expressing each gene; color scale indicates average expression level. (**G**) Cell type-associated cardiac marker expression across 8-day and 30-day ROIs, indicating shifts in the abundance of proliferative, fibroblast, endothelial, epicardium, and ventricular cardiomyocyte signatures over time.

To assess the temporal transcriptional dynamics of COs following *in vivo* implantation, we performed spatial transcriptomic profiling using GeoMx Spatial Transcriptomic (Figure 8A). The whole ROI transcriptomes were considered in the downstream analysis.

Differential gene expression analysis between Day 8 and Day 30 ROIs identified substantial transcriptional differentially expression, with a subset of genes significantly up (1472) and down (30) regulated comparing 30 days with 8 days after transplantation (Figure 8B and Table S8), where genes were top-upregulated (*ENHO, YPEL3, and KCNE1*), and top-down regulated (RPS11, HLA-DQB1, and OR11H1).

A heatmap of differentially expressed genes (DEGs) derived from spatial transcriptomic profiling of infarcted myocardial tissue at 8 and 30 days post-transplantation of COs was generated. Top 50 highly significantly upregulated genes at day 8 were significantly downregulated by day 30 (false discovery rate (FDR) < 0.05, log2 fold change > 1). Unsupervised hierarchical clustering revealed a distinct segregation of samples by time point, with a pronounced transcriptional activation at day 8 and a marked attenuation at day 30 (Figure 8C). Notably, the day 8 cluster exhibits upregulation of genes associated with autophagy and cell survival (e.g., *GABARAPL1 29*, *NOL3 30*), ECM remodelling and vascular support (*PDGFB 31*, *COL16A1 32*), ion channel activity (*KCNE1*, *NKX2-4*), and metabolic reprogramming (*ELOVL5*, *TTC19*).

Gene set variation analysis (GSVA) further demonstrated that Day 8 ROIs had significantly elevated enrichment scores for both fibrosis-related (*P* = 3.4 × 10⁻⁵) and TGF-β signalling pathways (*P* = 6.0 × 10⁻⁵) compared to Day 30 (Figure 8D - E). To further dissect this regulatory transition, we assessed the expression of TGF-β inhibitory components using GSVA and dot-plot analyses (Figure S17). ROIs (annotated for CMs, CFs, and ECs) from Day 30 exhibited significantly higher expression of endogenous TGF-β antagonists, including *FST*, *BMP2*, MMP2 and *MMP9*.

To evaluate the underlying cellular composition, we performed marker-based cell type comparison with published cardiac cell markers. ROIs from Day 8 exhibited expression of proliferative cells (*BRIC5TOP2A*), fibroblast (*POSTN, C7*), SMCs (NR2F2), pericyte (ACTN4), and adipocyte (KLF5) markers. In contrast, Day 30 ROIs showed a shift toward expression of cardiomyocyte (*ACTN2***,** *MYL4*), ECs (*PECAM1, NPR3*), epicardium (*TCF21*), and proliferative cardiac cells (*CENPE, DEPDC1*) markers (Figure 8F - G).

## Discussion

Cardiac cell replacement therapy aims at restoring cardiac function, especially following myocardial infarction. The success of the therapy critically depends on the suitable cell product. In this study, we describe a novel protocol that generates COs composed of the major types of myocardial cells: CMs, CFs, and ECs providing a platform technology to manufacture transplantable cMTs. A key innovation of the present protocol is the successful transfer from a 96-well-based approach of CO formation towards a scalable approach in stirred bioreactors. We provide evidence that COs generate in bioreactor culture efficiently engraft in a porcine model of acute MI, improve cardiac function and do not cause arrhythmia.

The concept of cardiac cell replacement therapy by intramyocardial cell transplantation with a putative reparative potential was tested in various settings in the last 30 years. Different cell types including myoblasts and cells of the hematopoietic lineage were proposed, as well as cell preparations of the foetal heart. The hypothesis that cells of non-cardiac origin can undergo transdifferentiation and thereby regenerate the heart has been postulated. If this hypothesis is valid, then the transplantation of cells from one part of the body to another could be a simple procedure that would effectively treat cardiac conditions. However, the inability of subsequent studies to reproduce the prior data and the observation of cell fusion instead of trans-differentiation, contradict this hypothesis 33.

The improvement in pluripotent stem cell technologies and finally the development of iPSCs opened new opportunities for cell replacement therapy, including the heart. However, transplanting monocultures of CMs derived from murine and human stem cells into different model animals demonstrated a low cell engraftment. In a previous study of our group, the retention of transplanted embryonic stem (ES) cell-derived CMs in a murine model was as low as 0.8% one day after transplantation 34. Ottersbach and colleagues further confirmed this outcome 35. Other investigators reported a moderate cell survival (4.2 ± 1.1%) four weeks after transplantation into acutely infarcted porcine hearts 36.

The issue of low CMs engraftment could by partially overcome through large scale cell transplantation, but this is limited by the scalability of cell production and the variation between batches 37. Conversely, the effects of dying cells after transplantation have not been fully analysed, and negative effects cannot be ruled out. Therefore, increasing the mass of transplanted cells to solve low engraftment should be viewed with caution.

Several studies suggest that increasing the engraftment of iPSC-CMs and thereby potentially improving the efficiency of cardiac cell therapy requires supplementation with additional other cell types 34, 38-40. Addition of embryonic fibroblast, e.g., enhances the engraftment of murine ES-CMs into murine myocardium 41. We verified these findings *in vitro* showing the potential of fibroblasts and mesenchymal stem cells (MSCs) to enhance the engraftment of CMs into avital cardiac tissue slices 39. Based on these findings a method to prepare aggregates of iPSC-CMs and MSCs was established for use in a murine model of cardiac cell therapy resulting in an increase of CM retention by one order of magnitude one day after transplantation 34. A substantial pitfall of the method was the lack of scalability. Therefore, a scalable method to generate cMTs for cell therapy is required. To generate cMTs the specific cardiac cell types were generated individually and combined to cMTs by different methods. One such method was based on spinning of cell suspensions in microtiter plates to arrange the cells into spherical MTs 42. Another approach was based on microfluidics 43. Although these strategies enable precise control of cell composition, they lack scalability.

To overcome the limited scalability, fine-tuning of iPSC differentiation could be part of the solution. This strategy involves developing a technology that can produce the three main cardiac cell types (CMs, ECs and CFs) in the form of COs in a single differentiation step. Current CO formation approaches rely on forming COs in microtiter plates, e.g. 96-well plates, to produce a uniform material. Apart from the lack of scalability, some recent protocols generate COs containing non-cardiac cells.

Our novel approach is based on the formation of COs by combined induction of CMs and ECs from spheroids of early mesodermal progenitors, which were generated from single cell suspensions of hiPSC, and by stimulating Wnt signalling. In an earlier study, we have described that biphasic block of Wnt signalling increases the stability of CM induction 13. We have combined the aforementioned approach with an optimized protocol to derive ECs from hiPSCs and generated COs composed mainly of CMs, CF, ECs, along with other cardiac cell types. Crucially, these COs do not contain any pluripotent stem cells or pacemaker cells at day 20 of differentiation.

After only 20 days of differentiation, the COs exhibit some features of maturation, as evidenced by adult-like sarcomere length, mature-like action potential shape and ultrastructural organisation. Single-cell RNA sequencing revealed elevated expression of key cardiac maturation markers, such as RYR2 and PLN. Functionally, COs displayed spontaneous and synchronised contractions, responding appropriately to physiological stimuli, including adrenaline and KCl. A mature CM phenotype was not achieved as indicated by remaining spontaneous contractions, a maximum diastolic potential that did not reach the expected value of -85 to -90 mV and a moderate upstroke velocity of about 14 V / sec.

At the preclinical level, this study demonstrates that COs successfully engraft and survive in the injured myocardium of a translational porcine model of acute MI, without inducing malignant arrhythmic events, and with potential beneficial effects on cardiac function and scar reduction. New cardiac cell therapies raise concerns on its potential proarrhythmic effects. Extended Holter monitoring and programmed electrical stimulation revealed that CO did not increase life-threatening arrhythmic events compared to controls. Remarkably, ventricular arrhythmic events were related to increased automaticity rather than scar-related re-entry and were confined to the early post-operative phase (< 48 hours) 44, 45.

Furthermore, previous large-animal studies have demonstrated a dose-dependent risk of arrhythmia following transplantation of pluripotent stem cell-derived CMs. Chong *et al*. reported that intramyocardial delivery of approximately 1 × 10⁹ human embryonic stem cell-derived CMs resulted in extensive remuscularization, but was accompanied by spontaneous ventricular arrhythmias during electrocardiographic follow-up 46. In contrast, Ye *et al*. employed a markedly lower cell dose (approximately 1 × 10⁷ hiPSC-derived CMs) in a porcine model of acute MI and observed no spontaneous or programmed electrical stimulation-induced arrhythmias, suggesting that reduced cell burden and supportive paracrine mechanisms may contribute to improved electrical stability 36.

In the present study, intramyocardial delivery of 3,500 COs corresponded to a total cell dose of 1.35 × 10⁸ ± 3.62 × 10⁶ cardiac cells, representing an intermediate dose relative to prior reports and 13.5-folds higher than reported by Ye and colleagues 36. Notably, no spontaneous arrhythmias were detected during ECG follow-up. While the absence of arrhythmia may partially reflect a moderately effective CM load, the organoid-based approach provides a structured multicellular microenvironment and paracrine signalling that may further promote electrical stability. Nevertheless, systematic dose-escalation studies with larger cohorts will be required to fully delineate the relationship between CO dose and arrhythmogenic risk, as well as whether COs excrete suitable transplantation supportive paracrine factors and/or cytokines.

Immunohistofluorescence analysis confirmed the presence of transplanted CO-derived cells in the myocardium at both 8- and 30-days post-transplantation. The initial detection of intact COs within the myocardium, followed by their progressive disaggregation into individual cells, suggests that COs integrate into the host tissue over time. The presence of HNA^+^ /cTnI^+^ and HNA^+^/vWF^+^ cells in the infarcted and border regions at 30 days indicates differentiation and possible contributions to CM and EC populations. These findings align with previous studies reporting that stem cell-derived cardiac grafts undergo structural remodelling and integration within the host myocardium 47. Further support for the therapeutic potential of CO transplantation came from CMR, which demonstrated significant improvements in LVEF, reduction in infarct size, and increased cardiac output after 30 days, along with trends towards increased stroke volume and left ventricular mass. Although the precise mechanisms underlying these benefits are yet to be fully elucidated, they are likely to involve a combination of direct functional contributions from engrafted cells and paracrine signalling that promotes angiogenesis, CM survival and ECM remodelling.

Spatial transcriptomic profiling of areas containing transplanted human COs revealed a significant reduction in the GSVA score for pro-fibrotic signalling (including *TGFB1*, *TGFB2*, *TGFB3*, *CTGF*, *COL1A1*, *POSTN*) at 30 days compared to 8 days. This supports the hypothesis that transplanted COs are not predisposed to adopt a fibrotic fate after transplantation into acutely injured myocardium, even though the host myocardium progresses toward scar tissue formation. Consistently, collagen quantification using Picrosirius Red staining suggested a potential anti-fibrotic remodeling in CO-treated animals compared to controls, characterized by lower collagen type I content and higher collagen type III content, further supporting a paracrine-mediated modulation of ECM remodeling. This study has some limitations. The relatively small sample size, particularly in the short-term groups, reduces the statistical power of certain analyses. Additionally, in-depth mechanistic investigations into electrical coupling and the long-term fate of cells post-transplantation were beyond the scope of this study. Future studies should address these aspects, as well as potential immune responses in the host.

In conclusion, we present a robust, scalable, and transportable platform for the generation of human COs mainly composed of CMs, ECs, and CFs. These COs exhibit structural, transcriptional, and electrophysiological hallmarks of functional CMs within COs, and retain pharmacological responsiveness even after non-cryogenic overseas shipment. Upon transplantation into a porcine model of acute MI, COs engrafted, dispersed within the injured myocardium, and improved cardiac function without provoking malignant arrhythmias. Our approach overcomes key translational bottlenecks in cardiac cell therapy - namely, poor engraftment, arrhythmogenic risk, persistence of pluripotent cells, presence of non-cardiac cells and lack of production scalability. These findings establish COs as a versatile platform for regenerative medicine, offering a pathway towards clinically relevant cardiac tissue repair.

## Materials and Methods

### HiPSC culture

hiPSC line NP0040-8 (kindly provided by Dr. Tomo Saric, Institute for Neurophysiology, Medical Faculty, University of Cologne, Germany) was cultured on Matrigel matrix (hESC-qualified, Corning, # 734-1440) pre-coated six well plates at 10 μg / cm^2^ growth area in E8 medium 14. The detail of optimal passaging, confluency controlling, and single cell suspension preparing was performed as previously described with the difference that E8 medium was additionally supplemented with 8.5 mM sodium bicarbonate (Sigma-Aldrich, #S6297) in this study 14, 19.

### Generation of hiPSC-CO

HiPSCs were dissociated into single cells when the culture reached 70-80% confluency. In order to ensure reproducibility, the hiPSC cultures used for COs production were maintained at above 95% pluripotency (determined by flow cytometry for SSEA4 and SSEA5). The CO induction was performed at two different scales: (1) Small scale production using U-shape bottom ultra-low attachment 96-well plate culture, and (2) Large scale production using suspension bioreactors culture (Applikon, My Control equipped with mass flow controllers and 250 mL multi-use glass vessel). Detailed information is depicted in Supplementary information section.

On day 0, 70-100 embryoid bodies (EBs) / mL were re-suspended into basal differentiation medium (see supplementary information for details) and supplemented with 12 μM CHIR99021 (LC Laboratories, # C-6556) for 24 hours. On day 1, medium was refreshed completely without CHIR99021 supplementation. On day 2, basal differentiation medium was also half refreshed and supplemented with 10 μM IWP2 (Tocris, # 3533), 10 μM XAV939 (Sigma-Aldrich, # X3004), 10 ng / mL human VEGF A165 (Peprotech, # 100-20), 10 ng / mL bFGF (Peprotech # 100-18B), and 0.2 mM 8-Bromoadenosine 3′,5′-cyclic monophosphate sodium salt monohydrate (8Bro) (Sigma-Aldrich, # 858463). EBs were kept on a rocking table at 40 reversions per minute (rpm) for 48 hours. On day 4, a complete medium change with 10 ng / mL VEGF, and 10 ng / mL bFGF was performed for 72 hours. On day 7 and onwards every three days medium was refreshed completely into maturation medium (M-Medium), prepared from Advance DMEM:F12 (**Table S9**).

From day 0 to the end of the experiment, 100 μg/mL ascorbate (Wako, # 013-12061) were continuously added to the media. COs were characterized from day 10 onwards. To that end, the COs were firstly washed with Dulbecco's Phosphate-Buffered Saline without calcium and magnesium ions (DPBS (-/-)) to remove any residual media and subsequently, on a shaker set to 40 rpm at 37 °C, 5 mL of 0.25% Trypsin-EDTA were added during 150 minutes for COs on day 20 with pipetting every half an hour. After that, 5 mL of DMEM:F12 was added to inactivate the trypsin. The resulting cell suspension was passed through a 40 μm cell strainer (Greiner Bio-One, #542040) to remove larger cell aggregates. The filtered cell suspension was then centrifuged at 300 x g for 2 minutes. Finally, the cell pellet was re-suspended in an appropriate volume of M-Medium for further use.

### Characterization of hiPSC-CO

Bright field images from COs were obtained from early starting time point (Day -2) to the end of the experiment using Nikon Eclipse Ts2. The size of the COs was measured using NIS Elements Software Version 5.21.03.

Whole mount and sliced COs were immunostaining against CD31 (Abcam, #ab28364), α-actinin (ACTN2) (Sigma-Aldrich, # A7811) and Anti-Connexin 43 (CX43; Sigma Aldrich, #c6219) as primary antibodies. Nuclei were counterstained with Hoechst 33342 (Sigma Aldrich, #14533). Finally, confocal fluorescent microscopy (TCS SP8, Leica Microsystems) was used for obtaining images.

To detect myosin bundles and sarcomere length measurements, COs were fixed with paraformaldehyde 4% at day 20 for 30 minutes at RT and embedded into 100 μL DPBS (-/-) inside cavity of slide (Carl Roth, # 1320102). Second-harmonic generation (SHG) images were acquired using a multiphoton microscope (TCS SP8 MP-OPO, Leica Microsystems) equipped with a tuneable Ti:Sapphire laser (Chameleon Vision II, Coherent). The laser was tuned to 880 nm and the SHG signal was detected with a non-descanned HyD detector with a BP filter 440 / 20. To evaluate CM maturation, the distance between myosin bands were measured via LAS X Office software to, and data were analysed by GraphPad Prism v6 (GraphPad Software, San Diego, California, USA).

For CO ultrastructural analysis, ultrathin sections of 70 nm of COs fixed from day 19 to 40 were cut using an ultramicrotome (Leica Microsystems, UC6) and a diamond knife from Diatome (Science Services # DU3530) and stained with 1.5% uranyl acetate (Agar Scientific, # R1260A) for 15 min at 37 °C and 3% Reynolds lead citrate solution made from Lead (II) nitrate (Roth, # HN32.1) and tri-sodium citrate dehydrate (Roth #4088.3) for 2 min. Images were acquired using a JEM-2100 Plus Transmission Electron Microscope (JEOL) operating at 80 kV equipped with a OneView 4 K camera. Detailed information is depicted in Supplementary Data section.

After COs dissociation, 0.25 x 10^6^ single cells were incubated with 1:50 of TNNT2-FITC (Miltenyi Biotec, # 130-119-575) according to antibody manufacture's protocols. Prior analysis flow cytometer compensation was performed to overcome spillover of fluorochromes, and data was acquired by a flow cytometer (LSR Fortessa Analyzer, BD Biosciences). FCS express 6 (De Novo Software, Glendale, CA) was used for evaluating data. Further details can be found in supplementary information section.

### TUNEL assay of apoptosis determination

In preparation for the TUNEL assay, day 100 and 213 COs 8 µm cryosections were incubated with TUNEL-Assay-Kit (ThermoFisher Scientific, Invitrogen, #C10617) and nuclei were counterstained with Hoechst 33342. Finally, the sections were examined by confocal fluorescent microscopy (SP8, Leica) and the obtained images were analysed with CellProfiler (Broad Institute of MIT and Harvard) to quantify the TUNEL positive cells. Positive (induced apoptosis through DNase application to CO slice prior to TUNEL assay), and negative (reaction cocktail without the fluorophore containing component) controls were also included in the analysis.

### Single cell RNA sequencing

From both 96-well plate and bioreactor systems, COs were transferred to Singleron company for single cell RNA sequencing (scRNA-seq; Cologne, Germany). Single cell dissociation and single cell RNA sequencing were performed as a service by Singleron (Cologne, Germany). All detailed information is depicted in Supplementary information section. RNA quality metrics are summarized in **Table S10**.

### Sharp electrode action potential measurement

Intracellular action potential (AP) measurements with sharp glass microelectrodes (20 - 50 MΩ resistance when filled with 3 mol l^-1^ KCl; World Precision Instrument, Sarasota, USA) have been performed as described before 48, 49. APs of CMs within the COs were measured at day 11, 21, and 27. Spontaneous contracting CM were measured without external stimulation. Inactive CMs were stimulated with a SD9 square pulse stimulator (Grass Technologies, West Warwick, RI, USA) using a unipolar custom-made stimulation electrode. After the first stimulation bursts, COs started to contract spontaneously again. Measurements were performed 30 min after the stimulation. The recording electrode was connected to an SEC-10LX amplifier (npi electronic, Tamm, Germany), and the signal was acquired with the Pulse software (HEKA, Lambrecht/Pfalz, Germany). AP amplitude, AP duration, AP duration at 50% of polarization (APD50), AP duration at 90% of repolarization (APD90), upstroke velocity, maximal diastolic potential (MDP, mean diastolic potential,) and the frequency of contracting were analysed with Mini Analysis (Synaptosoft, Fort Lee, USA). GraphPad Prism v8 was used for all statistical calculations and creating AP graphs.

### Optical mapping

COs were incubated for 15 min with a membrane potential-sensitive fluorescent dye (1 µM DI-4-ANEPPS) and Blebbistatin (10 µM) to remove motion artefacts. After, COs were placed in the optical mapping setup and superfused with Tyrode's solution (36 ± 0.2 ˚C) containing (in mM): NaCl 140, KCl 5.4, CaCl2 1.8, MgCl2 1.0, glucose 5.5, and HEPES 5.0 (pH 7.38). Optical APs were recorded using a CMOS camera (MICAM Ultima 100 × 100 pixels, SciMedia USA Ltd) and analysed using custom-made software 50.

### Electrocardiographic analysis of the COs

High-density microelectrode array (HD-MEA) was used to perform electrophysiological recordings of the extracellular field potentials of 30-day COs. The BioCAM DupleX platform (3Brain AG, Pfäffikon SZ, Switzerland) was paired with single-well CorePlate™ 38 / 60 / 90 3D HD-MEAs (3Brain), which comprise 4,096 gold-coated 3D electrodes arranged in a 64 × 64 grid with a 60 μm pitch, covering an active recording area of 3.8 × 3.8 mm.

Baseline spontaneous electrical activity of the CO in M-Medium was recorded for 4 minutes to establish control conditions. To assess the organoid's electrophysiological responsiveness and its ability to sustain synchronized electrical activity, 5 µM adrenaline was administered to the medium, and the activity of the COs was monitored for 5 minutes. Subsequently, without removing the adrenaline, 0.1 mM KCl was added to the medium, and the electrophysiological responses were monitored for an additional 5 minutes. All recordings were performed at 37 °C, and the chip was covered with a black lid to minimize light interference during data acquisition.

Cardiac field potentials (CFP) were detected and analysed with the algorithms available in BrainWave 5 (3Brain AG, Pfäffikon SZ, Switzerland). Parameters assessed included CFP rate; inter CFP interval; CFP minimum amplitude, and CFP maximum amplitude. All traces, plots, graphs, and videos were directly exported from BrainWave 5. Additional information is depicted in Supplementary information section.

### Porcine model of myocardial infarction

All experimental animal protocols were approved by the Animal Experimentation Ethical Committee Unit of the Germans Trias i Pujol Health Research Institute and Government Authorities (Generalitat de Catalunya; Code: 11208 and 12540), in accordance with the guidelines from Directive 2010 / 63 / EU of the European Parliament on the protection of animals used for scientific purposes or the NIH Guide for the Care and Use of Laboratory Animals 51. Animal Research: Reporting of *in vivo* Experiments (ARRIVE) reporting guidelines were used 52.

Thirteen crossbreed Landrace X Large White pigs (33.1 ± 2.6 Kg) (7 females) were sedated with an intramuscular (IM) injection of dexmedetomidine (0.03 mg / kg; Dexdor®, Orion Pharma, Espoo, Finland), midazolam (0.3 mg / kg; Laboratorios Normon, Barcelona, Spain), and butorphanol (0.3 mg / kg; Butomidor®, Richter Pharma AG, Wels, Austria). Then, anaesthetic induction was performed with an intravenous (IV) bolus of propofol (2 mg / kg; Propovet®, Zoetis, Barcelona, Spain). After, animals underwent endotracheal intubation, and anaesthesia was maintained by 2% isoflurane (IsoVet®, BBraun), inhalation. Fentanyl (0.075 mg / kg / 20 - 30 minutes, IV; Fentadon®, Dechra, Bladel, The Netherlands) and a 1.5 mg / kg atracurium besylate bolus (Sanofi Aventis S.A., Barcelona, Spain) were given during intervention. After a left lateral thoracotomy in the fourth intercostal space, acute MI was induced by a double-ligation (Optilene 5 / 0 W - 8556 12 - S, Ethicon Inc., Sommerville, NJ) of the first marginal branch of the circumflex artery, 1.5cm distal from the atrioventricular groove.

After a left lateral thoracotomy in the fourth intercostal space, acute MI was induced by a double-ligation (Optilene 5 / 0 W - 8556 12 - S, Ethicon Inc., Sommerville, NJ) of the first marginal branch of the circumflex artery, 1.5 cm distal from the atrioventricular groove. Thirty minutes after MI induction, each treated animal received a total of 3,500 COs suspended in 1.0 - 1.5 mL of Plasmalyte, administered via 5 - 6 intramyocardial injections using an 18 G needle. Control animals received 1.0 mL of Plasmalyte delivered through the same number of injections and using the same technique. The dose administered to each pig was determined by the bioreactor's total production capacity, which yielded approximately 7,000 COs per batch in a 250 mL vessel. For the initial assessment of safety and feasibility, this batch was split into two equal doses of 3,500 COs each.

Tulatromicin (2.5 mg / kg, IM; Draxxin®, Pfizer Animal Health, Madrid, Spain) was administered at the end of the surgery as antibiotic therapy and a transdermal fentanyl patch was applied to allow analgesic post-operative care (Durogesic®, Janssen-Cilag, Madrid, Spain).

All animals were immunosuppressed with 250 mg of methylprednisolone (Sanofi Aventis S.A.) on day of CO transplantation followed by 125 mg per day until euthanasia, and cyclosporine A (10 - 16 mg / kg, oral once per day; Neoral®, Novartis, Madrid, Spain), 3 days before surgery until end of follow up. Additionally, the day of surgery and 15 days after MI induction, animals received an IV dose of abatacept (12.5 mg / kg; Orencia®, Bristol-Myers Squibb, Munich, Germany), as previously described 53. Animals were randomized as: Long-term Control (n = 4; 30 days of follow up), Long-term CO (n = 7; 30 days of follow up), and Short-term CO (n = 2; 8 days of follow up) groups. Finally, after 8 or 30 days of follow up, animals were euthanized through a pentobarbital sodium overdose (200 mg / kg, IV; Dolethal®, Vetoquinol E.V.S.A, Madrid, Spain).

### Cardiac electrocardiographic monitoring

After surgery, pigs were continuously monitored with a 2-channel ECG external Holter recorder (AFT - 1000, Holter Supplies, Paris, France) for 15 days in the Long-term groups. Cardiac arrhythmias such as ventricular premature contractions (VPC non-sustained (NSVT) or monomorphic sustained ventricular tachycardia (MSVT) or ventricular fibrillation (VF) were recorded.

### Cardiac magnetic resonance

Long-term animals (Control, n = 4; and Long-term CO, n = 7) underwent to 2 cardiac magnetic resonance (CMR) exams at 2- and 30-days post-MI induction using a 3T imaging system (Vantage Galan 3T, Canon Medical Systems, Otawara, Tochigi, Japan). Cine, flow, mapping and late gadolinium-enhanced (LGE) CMR sequences were acquired. Left ventricular (LV) and right ventricular (RV) volumes, mass and ejection fraction (EF) were measured on cine images from the stack of short-axis slices covering the ventricles from base to apex. Volumes and LV mass were indexed for body surface area. Cardiac output and index were calculated from the LV stroke volume (SV) derived from the aortic flow. Pre- and post-contrast T1 and pre-contrast T2 mapping sequences were acquired to assess diffuse fibrosis and myocardial oedema respectively. Myocardial extracellular volume (ECV) was derived from the pre- and post-contrast T1 mapping values adjusted for haematocrit at time of CMR. The amount of LGE (scar) was assessed using the full-with half-maximum (FWHM) methods. Both, total grams of LGE and percentage of LGE over total myocardial mass were calculated. All cardiac MRI parameters were analysed with Medis Medical Imaging software (Leiden, Netherlands). Detailed information is depicted in supplementary information section.

### Electroanatomical high-density mapping

Long-term control and CO animals underwent an electroanatomical mapping and electrophysiological study (EPS) at day 30 (before euthanasia), to characterize the electrophysiological properties of the scar and to test ventricular arrhythmias inducibility. Invasive endocardial LV high-density mapping (HDM) was carried out via retrograde aortic access using the Rhythmia HDx 3D Mapping System (Boston Scientific, Marlborough, Massachusetts, USA) and a 64-pole basket catheter (INTELLAMAP ORION, Boston Scientific, Marlborough, Massachusetts, USA). HDM was performed at a paced cycle length of 400 ms with 1 extrastimuli 20 ms above the ventricular effective refractory period. Filling threshold was ≤ 2 mm in regions of low bipolar voltage and ≤ 10 mm elsewhere; interpolation between points ≤ 2 mm was required in all cases. Bandpass filters were set at 30 to 300 Hz for bipolar signals and 1 to 300 Hz for unipolar signals.

### Myocardial tissue collection for histological and immunohistofluorescence analysis

After mid-sternotomy, hearts were excised and washed in PBS, then sliced transversely into three 1 to 1.5 cm sections from the ligation to the apex and digitally photographed. Next, 5 mm tissue transverse samples from the infarct core, border zone, and the septum noninfarcted wall (distal myocardium) of each section were obtained, fixed in 10% neutral buffered formalin, and embedded in paraffin, or frozen in an optimal cutting temperature compound (OCT) for histopathologic and immunohistofluorescence analyses, respectively.

On 4 µm-thick paraffin-embedded sections, Masson's trichrome and Picrosirius Red staining was done for the primary histological examination, and fibrosis modulation assessment, respectively. Picrosirius Red-stained slides were captured under polarized light with an Axio Observer Z1 microscope (Zeiss, Oberkochen, Germany). Then, 4 to 5 representative 750000 μm^2^ regions of interest (ROIs) were selected and further quantified using Image-Pro Plus software (v.6.2.1; Media Cybernetics, Inc).

Immunohistofluorescence was carried out on 10 µm-thick OCT snap-frozen slices. Briefly, sections were incubated with cardiac troponin I (cTnI) (1/100; Abcam, Cambridge, UK), human nuclei antigen (HNA) (1 / 50; Millipore, CA, USA), CD31 (1 / 200, Abcam), and von Willebrand Factor (vWF) (1 / 100; Abcam) primary antibodies, and Alexa Fluor 488, 594, 647 and Cy3 (1 / 500; Jackson ImmunoResearch Laboratories, Cambridgeshire, UK) secondary antibodies. Finally, nuclei were counterstained with 4′,6-diamidino-2-phenylindole dihydrochloride (DAPI) (1 / 1000) (Sigma-Aldrich, Madrid, Spain), and sections were analysed under an Axio Observer Z1 microscope (Zeiss, Oberkochen, Germany) and Leica Stellaris 8 confocal microscope with White Light Laser (Leica Microsystems, Wetzlar, Germany).

### Spatial transcriptomics using GeoMx DSP

OCT-embedded tissues collected at 8 (Short-term CO) and 30 days (Long-term CO) post-transplantation were sectioned and mounted onto GeoMx spatial transcriptomic slides. A total of 36 ROIs were selected for spatial transcriptomic library preparation, comprising 19 ROIs from the 8-day and 17 ROIs from the 30-day post-transplantation samples. Human cells within the ROIs were identified based on area-of-interest (AOI)-specific detection positive for HNA. R program version (4.3.2) and RStudio version (2023-12.0.369) were used for downstream data analysis of count matrix and metadata gene expression analysis. Additional methodological details are provided in the supplementary information.

### Statistical analysis

De Novo software version FCS Express 6 was used for flow cytometry analysis. GraphPad Prism software version 5 was used for graph drawing and statistical analysis. SPSS software was used for CMR, ECG-Holter and HDM analyses. *In vitro*, to find statistical difference between groups, one-way analysis of variance (ANOVA) was used, and Bonferroni's test also was used as a post hoc test. Data were represented as mean ± standard deviation (mean ± SD) when biological independent replications was three to six (n = 3 - 6) and significant difference value was less than 0.05 (P < 0.05). All data from AP measurements are presented as mean ± SEM. Differences of AP parameters were tested for statistical significance by Student's *t*-test and one-way ANOVA analysing P for trend. A two-sided *P*-value < 0.05 was considered statistically significant. *In vivo*, paired t-test was used to assess changes in CMR data over time and within groups. Student's *t*-test or Chi-square were applied to assess differences between groups in ECG-Holter monitoring and HDM results. Data were expressed as mean ± SD. Q - Q plot was used for assessing data distribution and normality.

## Supplementary Material

Supplementary materials and methods, figures.

Supplementary tables.

Supplementary movie 1: MWB-Lab_96Well-hiPSCs-Cardiac-Organoids_Day7.

Supplementary movie 2: MWB-Lab_Bioreactor-hiPSCs-Cardiac-Organoids_Day7.

Supplementary movie 3: MWB-Lab_hiPSC-Cardiac-organoids_On_HD-MEA.

Supplementary movie 3: MWB-Lab_hiPSC-Cardiac-organoids_On_HD-MEA.

## Figures and Tables

**Table 1:** ** ECG-Holter data.** Electrocardiogram Holter monitoring data for Short-term CO, Long-term Control, and Long-term CO groups. For Long-term groups, statistical significance indicated according to Student's t-test for independent measures. Data are presented as mean ± SD. HR indicates heart rate; PVC, premature ventricular contractions; NSVT, non-sustained ventricular tachycardia; MSTV, monomorphic sustained ventricular tachycardia; AF, atrial fibrillation; AVB, atrioventricular block**.** Early; 0 to 48 hours post myocardial infarction, and Late; 48 hours to 15 days post myocardial infarction. P values: ≤ 0.05 is considered as Significant.

	Long-term Control (n = 4)	Long-term CO (n = 3)	P value
Mean HR (bpm)	101 ± 11	102 ± 4	0.953
Min HR (bpm)	53 ± 16	63 ± 1	0.316
Max HR (bpm)	258 ± 1	221 ± 44	0.275
PVC count	26684 ± 20787	57917 ± 36370	0.205
NSVT early (< 48h)	100%	100%	-
NSVT late (≥ 48h)	0%	33%	0.212
MSVT early (< 48h)	75%	100%	0.350
MSVT late (≥ 48h)	0%	0%	-
AF early (< 48h)	50%	67%	0.659
AF late (≥ 48h)	0%	0%	-
AVB	25%	0%	0.350

**Table 2:** ** Cardiac Magnetic Resonance Imaging.** CMR data at 2- and 30-days post-MI for Long-Term Control and CO-treated animals. Data distribution was assessed using visual inspection of quantile-quantile (Q-Q) plots. Statistical significance indicated according to paired Student t-test within each group. Data are presented as mean ± SD. Significant P values (≤0.05) are indicated in bold. iLVEDV indicates indexed left ventricular end-diastolic volume; iLVESV, indexed left ventricular end-systolic volume; LVSV, left ventricular stroke volume; LVEF, left ventricular ejection fraction; iLV Mass, indexed left ventricular mass; iRVEDV, indexed right ventricular end-diastolic volume; iRVESV, indexed right ventricular end-systolic volume; RVSV, right ventricular stroke volume; RVEF, right ventricular ejection fraction; LGE, late gadolinium-enhanced; FWHM, full width half maximum; ECV, extracellular volume; Ao, aortic; LV, left ventricular.

	Long-term Control (n = 4)			Long-term CO (n = 5)		
	2d post-MI	30d post-MI	P value	2d post-MI	30d post-MI	P value
iLVEDV (mL)	98.4 ± 10.1	90.0 ± 17.2	0.278	98.3 ± 15.1	91.2 ± 7.9	0.317
iLVESV (mL)	57.8 ± 14.9	46.7 ± 14.5	0.176	59.1 ± 17.2	41.8 ± 8.8	0.056
LVSV (mL)	28.0 ± 6.8	33.4 ± 6.4	0.459	28.4 ± 5.5	41.1 ± 11.1	0.050
LVEF (%)	41.6 ± 11.1	48.6 ± 9.0	0.448	40.5 ± 9.1	53.8 ± 10.7	0.034
iLV Mass (gr)	79.7 ± 10.1	81.8 ± 11.0	0.711	69.2 ± 4.1	75.9 ± 10.9	0.242
iRVEDV (mL)	82.0 ± 31.6	67.7 ± 11.1	0.400	93.9 ± 23.2	76.6 ± 15.3	0.078
iRVESV (mL)	50.3 ± 19.1	36.8 ± 13.4	0.194	45.6 ± 18.7	35.8 ± 13.5	0.260
RVSV (mL)	21.8 ± 11.0	24.1 ± 10.4	0.838	35.0 ± 12.4	34.2 ± 12.7	0.657
RVEF (%)	37.3 ± 12.4	45.7 ± 18.3	0.603	51.3 ± 17.1	52.7 ± 17.3	0.740
Scar size as LGEmass (gr)	3.2 ± 1.9	2.9 ± 1.3	0.699	2.9 ± 0.8	2.3 ± 0.8	0.234
Scar size as percentage of LGEmass (%)	5.9 ± 3.4	4.8 ± 2.4	0.417	5.8 ± 1.9	3.7 ± 1.3	0.029
Native T1 mapping (ms)	1247.1± 123.8	1115.3 ± 30.1	0.114	1178.6 ± 33.7	1138.7 ± 68.6	0.154
ECV (%)	31.7 ± 7.3	24.1 ± 5.1	0.317	27.4 ± 3.0	21.1 ± 5.2	0.085
T2 mapping (ms)	41.8 ± 3.4	42.6 ± 3.4	0.419	46.5 ± 2.8	40.9 ± 3.9	0.112
Ao Forward flow (ml)	26.3 ± 15.2	29.2 ± 4.5	0.770	29.7 ± 1.9	36.9 ± 3.9	0.040
LV Cardiac output (L/min)	3.3 ± 0.8	3.4 ± 0.8	0.909	3.2 ± 1.2	5.0 ± 0.7	0.010
LV Cardiac index (L/min/m^2^)	4.8 ± 1.2	4.3 ± 0.9	0.616	4.4 ± 1.5	6.0 ± 1.2	0.048
Mitral regurgitation fraction (%)	14.8 ± 20.1	-1.2 ± 39.1	0.444	-0.9 ± 13.9	8.2 ± 27.0	0.460

**Table 3:** High-Density Electroanatomic Mapping. High-density electroanatomic mapping data at 30 days post-MI for Long-Term Control and CO-treated animals. Statistical significance indicated according to Student's t-test for independent measures. Data are presented as mean ± SD. mV indicates millivolts; VT, ventricular tachycardia.

	Long-term Control (n = 4)	Long-term CO (n = 5)	P value
Area ≤ 1.5 mV (cm^2^)	1.3 ± 0.3	0.5 ± 0.8	0.107
Area ≤ 0.5 mV (cm^2^)	0.4 ± 0.4	0.1 ± 0.3	0.260
Area ≤ 0.1 mV (cm^2^)	0	0	-
Area 0.5 - 1.5 mV (cm^2^)	0.9 ± 0.2	0.4 ± 0.6	0.113
Decelleration zones	0	0	-
VT inducibility	0	0	-
