# Supplementary material for: Scalable differentiation of human cardiac organoids from iPSCs generates cardiac tissues for cardiac cell therapy
Source: Theranostics. 2026 Apr 23;16(12):6437–62. doi: 10.7150/thno.127654 (PMC13231838; doi:10.7150/thno.127654)
Supplement: Supplementary file 1 — Supplementary materials and methods, figures. [file thnov16p6437s1.pdf]

Extended Materials and Methods, Figures and Tables with Legends

Generation of hiPSC-CO

Small scale of COs from hiPSCs production using U-shape bottom ultra-low attachment 96-well plate culture.

To generate individual COs, 0.025 X 10^6 hiPSCs in 5 µL E8 medium were added on the bottom of 100 µL E8 medium inside a well of a U-shape bottom ultra-low attachment 96 well plate. The plate was centrifuged at 2400 x g at 25 °C for 10 minutes, incubated at 37 °C with 5% CO_2 for 48 hours, and after 48 hours the singular spheroids (embryoid bodies, EBs) were transferred via a cut tip of a 1000 µL pipette into a well of a 12-well plate that contained 1 mL medium. Alternatively, 70 EBs / mL were pooled into a 3.5 cm dish and/or a well of six well plate that contained 2 mL medium. The dish / plate was cultured on a rocking table at 40 reversions per minute (rpm) at 37 °C with 5% CO_2.

Large scale of COs from hiPSCs production using suspension bioreactors culture

Using culture dishes, 0.3 X 10^6 / mL hiPSCs were added in E8 medium inside a well of a six-well plate. For larger scale aggregation, cells were cultured in 10 cm dishes containing higher cell numbers and agitated at 40 rpm at 37 °C with 5% CO_2 for 48 hours. From seeding (designated as day -2) to the start of mesodermal induction (day 0), E8 medium was supplemented with 5 µM ROCK, and the plates or dishes were incubated inside a humidified incubator at 37°C and 5% CO_2. Basal differentiation medium consisted of RPMI 1640 (Thermo Fisher Scientific, # 61870-010) with 1× B27 supplement without insulin (Thermo Fisher Scientific, # 175044) and 100 µg / mL ascorbate (Wako, # 013-12061).

Applikon 250 mL stirred suspension bioreactors were used for large-scale culture. The inner vessel walls were siliconized with 1 mL Sigmacote (Sigma-Aldrich, # SL2). Vessels were gently swirled to cover the whole surface with Sigmacote. Coated vessels were kept under the chemical fume hood for one hour to remove the residual amount of liquid Sigmacote. Bioreactor parts were assembled including: glass vessel, head caps, the gassing filters were covered. The vessel was autoclaved at 121°C for 15 minutes, and cooled to room temperature. Under a laminar airflow cabinet, 100 mL of E8 medium were added per 250 mL vessel. The vessel was placed back into the vessel holder. One-hour vessel equilibration was performed at 140 rpm stirring speed with overlay gassing containing 95% air, and 5% CO_2 at a total flow rate of three standard litres per hour (sL / h) at 37 °C. Once COs differentiation was finished, the bioreactor vessels were washed with distilled water and 70% ethanol.

hiPSC-COs immunofluorescence staining

Whole mount immunostaining**:** COs were transferred into a well of 48-well plates via cut tips of a 1000 µL pipette and the medium was discarded from the well. 4% PFA was used for CO fixation for 30 minutes at 4 °C, and 0.5% BSA with 0.5% Triton- X-100 was used for CO permeabilization for 30 minutes on shaker with 45-60 rpm at RT, as well as COs were blocked with 3% BSA in DPBS for

60 minutes on shaker with 45-60 rpm at RT. Blocking solution was removed, and 200 µL of 3% BSA was added containing 1:200 dilution ratio of CD31 (Abcam, #ab28364) and α-actinin (ACTN2) mouse monoclonal IgG1 (Sigma-Aldrich, # A7811) overnight on shaker with 45-60 rpm at 37 °C. After 24 hours, the well was washed two times with 3% BSA in DBPS (-/-), and 200 µL of 3% BSA was added containing 1:500 Alexa Fluor 555 goat α-rabbit (Life Technologies, # A21430), 1:500 Alexa Fluor 647 goat α-mouse IgG1 (Life Technologies, # A21241), and Hoechst 33342 for 60 minutes on shaker with 45-60 rpm at 37 °C. The plate maintained in the dark condition.

Sliced COs: For preparation of cryoslices, COs were fixed by 4% PFA. Fixed COs were embedded in Tissue-Tek (Sakura, # 1227900003) and stored frozen at -80°C, cryo-sliced at 8 µm thickness. Immunostaining protocol was applied similar to whole mount staining but on flat slides without applying agitations. Three washing steps were performed with 3% BSA in DBPS slowly, and confocal fluorescent microscopy (TCS SP8, Leica Microsystems) was used for obtaining images.

TUNEL Assay of apoptosis determination

In preparation for the TUNEL assay, COs were fixed in 4% PFA and frozen before being cryosectioned into 8 µm slices (CM3050 S Cryostat, Leica) and put on slides. Sections were circled with a PAP Pen (Agilent, #S2002) and fixed for 30 minutes at 37°C in 4% PFA. After washing the sections twice with 1X DPBS (-/-) they were incubated in Proteinase K solution (1:25 in 1X DPBS (-/-) diluted Component H, Click-iT Plus TUNEL-Assay-Kit; ThermoFisher Scientific, Invitrogen, #C10617) at 37°C for 15 minutes. Sections were washed with 1X DPBS (-/-), fixed again with 4% PFA for 5 minutes at 37°C and washed twice with 1X DPBS (-/-). To produce a positive control, one section was incubated in DNAse (ThermoFisher Scientific, Invitrogen, #18068015) in a concentration of 0.04 units / µl for 30 minutes at room temperature. TdT Reaction buffer (Component A, Click-iT Plus TUNEL-Assay-Kit; ThermoFisher Scientific, Invitrogen, #C10617) was added for 10 minutes at 37°C to the sections prior to incubation of the sections for 60 minutes in a humidified chamber at 37°C in TdT reaction mixture (Click-iT Plus TUNEL-Assay-Kit; ThermoFisher Scientific, Invitrogen, #C10617). For permeabilization, sections were incubated in 3% BSA and 0.1% Triton-X-100 in 1 x DPBS (-/-) for 5 minutes. The Click-iT Plus reaction was performed by incubating the sections in the Click-It Plus TUNEL reaction cocktail (Click-iT Plus TUNEL-Assay-Kit; ThermoFisher Scientific, Invitrogen, #C10617) for 30 minutes at 37°C.

Sections were then incubated for 5 minutes in 3% BSA in 1 x DPBS (- / -) before counterstaining the nuclei with Hoechst 33342 (Sigma Aldrich, #14533) 1:500 in 3% BSA in 1x DPBS (-/-) for 30 minutes at 37°C. After washing the sections twice with 1x DPBS (- / -) antifade mountant (ThermoFisher Scientific, #P36934) and coverslips were added. Finally, the sections were examined by confocal fluorescent microscopy (SP8, Leica) and the obtained images were analysed with CellProfiler (Broad Institute of MIT and Harvard) to quantify the TUNEL positive cells.

hiPSC-COs transmission electron microscopy

COs were fixed at day 19 to 40 in 2% glutaraldehyde (Sigma, # G5882-100ML) with 2.5% sucrose (Roth, # 4621.1) and 3 mM $CaCL_2$ (Sigma, # C7902-500G) in 0.1 M HEPES (Roth, # 9105.2) buffer pH 7.4 for 30 min at RT and overnight at 4°C. Samples were washed four times with 0.1 M sodium cacodylate (Applichem, # A2140,0100) buffer and incubated with 1% osmiumtetroxid (Science Services, # E19190) and 1% potassium ferricyanid (Sigma, # P8131) for 1 h at 4°C. After 4 x 15 min wash with 0.1 M cacodylate buffer, samples were dehydrated at 4°C using ascending ethanol series (VWR, # 153386F); (50%, 70%, 90%, 3 x 100%) for 15 min each step. The samples were incubated with a mix of 50% ethanol / propylenoxide (Sigma, # 8.07027.1000) and two times with pure propylenoxide for 15 min. Samples were infiltrated with a mixture of 50% Epon (Sigma, # 45359-1EA-F) / propylenoxide and 75 Epon / propylenoxide for 2 h each at 4°C and with pure Epon overnight at 4°C. The next day, Epon was exchanged and samples were incubated for 2 h at RT, placed into TAAB capsules and cured for 72 h at 60°C.

Ultrathin sections of 70 nm were cut using an ultramicrotome (Leica Microsystems, UC6) and a diamond knife from Diatome (Science Services # DU3530) and stained with 1.5 % uranyl acetate (Agar Scientific, # R1260A) for 15 min at 37°C and 3% Reynolds lead citrate solution made from Lead (II) nitrate (Roth, # HN32.1) and tri-sodium citrate dehydrate (Roth #4088.3) for 2 min.
Images were acquired using a JEM-2100 Plus Transmission Electron Microscope (JEOL) operating at 80 kV equipped with a OneView 4 K camera.

hiPSC-CO flow cytometry

COs were dissociated into single cells. They were fixated by 4% PFA for 15 minutes at room temperature, and permeabilized with 0.5% Triton X-100 in DPBS(- / -) for 15 minutes. The staining was performed with Cardiac Troponin T-FITC (Miltenyi Biotec, # 130-119-575) according to antibody manufacture´s protocols. For surface antibodies e.g., 1:50 dilutions of α-human-CD31-APC (Miltenyi Biotec, # 130–110-670), and α-human-VE-Cadherin-PE (Miltenyi Biotec, # 130–100-716) cells were directly stained according to manufacture protocols. Prior analysis flow cytometer compensation was performed to overcome spillover of fluorochromes, and data was acquired by a flow cytometer (LSR Fortessa Analyzer, BD Biosciences). FCS express 6 (De Novo Software, Glendale, CA) was used for evaluating data.

Single cell RNA sequencing

Ten COs at day 10 and 20 from 96-well plate system, ten COs at day 20 from bioreactor system, and 2 big organoids (about 4 mm) at day 133 from 96-well plate, separately were transferred into separate 15 mL Falcon tubes via cut tips of a 1000 mL pipette. The tubes were filled with M-Medium to remove air bubbles and directly transported at RT to Singleron company for single cell RNA sequencing (scRNA-seq; Cologne, Germany). Single cell dissociation and single cell RNA sequencing were performed as a service by Singleron (Cologne, Germany).

In brief, experimental workflow was as follows: 1- Single cell suspension preparation and quality inspection: Samples were processed to obtain single cell suspension. For tissue samples, sCelLiVETM Tissue Dissociation Buffer was used to dissociate tissues into a single cell suspension. Cell concentration and viability was determined to assess the sample quality. The tissue dissociation quality control results and remarks are based on Singleron's standard quality criteria. The classification requirements of the test results are cell number more than 20000 cells and the cell viability more than 85% viable cells. 2- Single cell isolation and labelling: The cells were loaded into the microfluidic SCOPE-chipTM, and were allowed to settle into the microwells. Following the removal of cells that did not settle into a well, beads containing unique cell identifying tags (Cell Barcodes) were flowed into the SCOPE-chipTM and allowed to settle in the wells on top of the cells. The cells were then lysed and the mRNA from each cell hybridized to the barcode sequences on the bead in the same well. 3- Reverse transcription of primed RNA into complementary DNA (cDNA) and cDNA amplification: The beads, now containing the hybridized mRNA from the cells, were collected from the SCOPEchipTM, and the hybridized mRNA was reverse transcribed into cDNA. The resulting cDNA was then amplified by a minimal number of PCR steps to generate sufficient material with which to create a sequencing library. 4- Preparation of single cell sequencing library: The resulting amplified cDNA was fragmented, ligated to adapters and PCR amplified to construct a sequencing library suitable for the Illumina sequencing platform.

<u>Data analysis:</u> An estimated cell number between 5000 to 6000 cells. The core data analysis process consists of five steps:

1- Data quality control by removing doublet and low-quality cells according to a certain threshold. To this end, CeleScope (CeleScope standard process and instructions can be found at the following link: https://github.com/singleron-RD/CeleScope) was used to generate a single-cell gene expression matrix file based from the raw sequencing data. Standard single-cell gene expression QC metrics (**Table S2**) were used to identify high quality single cells. Only high-quality single cells were used for downstream analysis. We focused on three key metrics for identifying high quality cells and removing poor quality ones: firstly, doublets are found by identifying cells with large gene or UMI counts; secondly, dead / dying cells are found by measuring mitochondrial RNA reads; and finally, debris cells were removed with small UMI counts. Data count matrixes, barcodes and RNA features of each COs samples were imported into the R program version 4.2.2 and Seurat v4 package was used (for details visit this link: https://github.com/satijalab/seurat)[54] to generate Seurat objects and then further data analysis, including sample alone and integration, log normalization data matrix, scaling data, data 2 dimension Principle component analysis (PCA) and Uniform Manifold Approximation and Projection (UMAP) reduction, as well as find neighbours, clusters, differentially gene expression between clusters and ontology gene expression biological pathways analysis.

2- Data processing: R-software with Seurat v4 package was used for down-stream data analysis. Log data normalization was performed, data was scaled, and the data dimensions were reduced by

PCA. (data, nPCA = 30), The first 30 dimensions were selected for cell clustering. Finally, UMAP dimension reduction analysis was performed for visualization.

3- Analysis of differential gene expression: The statistical Wilcoxon test was used to identify genes differentially expressed between clusters. Marker genes expressed in each individual cluster were compared to all remaining clusters, and only the differentially expressed gene markers were reported. All significantly, differentially expressed genes per cluster were separated per cluster, sorted by lowest p-value (p_val) from zero, and the top 10 differentially expressed genes assigned with lowest p-value per cluster were used to annotate cell types.

4- Manual annotation of cluster cell identity: Firstly, top 10 differentially expressed genes per each cluster were compared with two online databases: PanglaodDB[21] and "The human protein Atlas" single cells, for further confirmation of cell identity, cardiac cells markers were collected from published articles[18, 23-25]. Those markers were used to confirm cardiac cell types within the different clusters. Finally, both online data base evaluation and the assignment of cell-specific markers based on published articles were used to define the cell identity. Based on this evaluation the clusters were annotated with the respective cell identity.

5- Gene ontology enrichment analysis: After determining differentially genes differentially expressed between clusters by Wilcoxon-test, a list of genes was prepared by using avg_log2FC > 0.5 function. Next, the list of genes was used to determine gene ontology biological pathways by using software libraries in R (clusterProfiler, AnnotationDbi, Bioconductor (org.Hs.eg.db), Seurat). The gene list was prepared and the 30 top ranking gene ontologies were plotted to visualize biological pathways.

Electrocardiographic analysis of the COs

High-density microelectrode array electrophysiological data were analysed using BrainWave 5 software. Each squared electrode has a diameter of 12 µm and a hight of 90 µm, whereby only to top surface is conducting. All 3D electrodes sit on top of pedestals, which together form passive microfluidic channels that allow liquid exchange below the sample. Recordings at 20 kHz sampling frequency were acquired using the BrainWave 5 software. Field potential signals from beating COs were acquired through a 0.02 to 5 kHzbandpass filter, and electrode number 1.1 on the array was used as the reference electrode.

Before the recording, the CorePlate™ well was washed with PBS to clean its surface. Afterwards, the chip was completely dried before a single CO was carefully placed on the array with as little medium as possible, using a cut 1000 µL pipette tip. Once the proper location of the organoid on the chip was confirmed, the remaining liquid was removed. During the removal of the liquid, capillary forces pulled the COs onto the electrodes, ensuring a good penetration of the electrodes into the COs. Immediately afterwards, 1 mL of M-Medium was added. The stability of the waveforms was confirmed for a period of 5 minutes to ensure consistency before starting the recording.

Cardiac MR imaging

All cardiac MR imaging was performed in a 3T state-of-the-art imaging system (Vantage Galan 3T, Canon Medical Systems, Otawara, Tochigi, Japan) with all animals in prone position using a 16-element phased array coil (Atlas SPEEDER Body Coil) placed over the chest. Images were acquired during breath-holds with electrocardiographic gating. We used segmented k-space steady state precession cine sequences (typically TR/TE 3.0/1.5 ms, 65º flip angle, field of view (FOV) 291 × 265 mm, pixel matrix 352 × 320, slice thickness 8 mm, 1302 Hz / pixel bandwidth) at 2, 3, and 4 chamber and short axis views from base to apex with no gap. Flow phase-contrast sequences (typically TR/TE 5.4 / 2.6 ms, 10º flip angle, FOV 350 × 350 mm, slice thickness 8 mm, 130–200 cm / s VENC) were performed at the aortic sino-tubular junction to calculate the aortic forward flow. Pre and post contrast T1 mapping sequences (MOLLI) were acquired matching short-axis cine positions. T2 mapping sequences were acquired matching short-axis cine positions. Delayed enhancement images were acquired 10 to 20 min after IV injection of gadolinium-based contrast (Gd-DTPA; 0.2 mmol / kg) using a phase-sensitive inversion recovery sequence (TR/TE 8.9/3.4 ms, 20º flip angle, inversion time 180–250 ms, FOV 340 × 340 mm, pixel matrix 572 × 448, slice thickness 8 mm, 140 Hz / pixel bandwidth) matching cine image positions. Inversion time was optimized to null the normal myocardium.

All images were reviewed and analysed off-line using the CMR imaging (cMRI) dedicated analysis software (Medis Medical Imaging software (Leiden, Netherlands)) by a level 3 cMRI expert blinded to the clinical data. Left ventricular (LV) and right ventricular (RV) endocardial borders (papillary muscles excluded) were manually traced in all short-axis cine images at the end-diastolic and end-systolic frames to determine end-diastolic and end-systolic volumes (EDV and ESV, respectively), using a dedicated post-processing software (QMass, v.8.1; Medis Medical Imaging Systems, Leiden, the Netherlands). LV mass was calculated by subtracting the endocardial volume from the epicardial volume at end diastole and then multiplying by tissue density (1.05 g / mL). Left and right ventricular ejection fractions (LVEF and RVEF, respectively) were calculated. Aortic forward and regurgitant flow and regurgitant fraction was calculated from the flow phase contrast sequences using a dedicated post-processing software (QFlow, v.8.1; Medis Medical Imaging Systems, Leiden, the Netherlands) Background noise correction was performed on all images. The endocardial and epicardial contours on delayed enhancement images were also outlined manually. Regions of interest were then manually traced in the hyperenhanced area at the place of maximum signal intensity and in the normal-appearing remote myocardium. As previously described, the areas of hyperenhanced myocardium were then automatically segmented by using a full-width at half-maximum algorithm with QMass. Two corrections were required for all automated regions of interest. First, microvascular obstruction (defined as hypointensity within a hyperintense region in subjects with infarctions) was adjusted to be included as late gadolinium enhancement (LGE) if present. Second, any obvious blood pool or pericardial partial voluming and artefacts were further removed. Scar volume for each slice was calculated as scar area × slice thickness. The scar mass was expressed as total scar volume × 1.05 g. Scar size was also expressed as a percentage of the total myocardial volume: (scar volume / myocardium volume) × 100. T1 and T2 mapping sequences were

analysed using dedicated postprocessing software (QMap v2.2, Medis Medical Imaging, Leiden, the Netherlands)

Height, weight, and heart rate of the pigs was recorded at every cMRI scan. Volumes and mass were indexed by body surface area (BSA) of the animals calculated as previously described[55]. The cardiac index was calculated as (forward aortic volume × heart rate)/BSA.

Tissue collection

After mid sternotomy, excised hearts were washed in ice-cold saline buffered solution to remove any residual blood, sliced transversely into three 1 - 1.5 cm sections (S1 - S3) from artery ligation to the apex, and digitally photographed.

Tissue transverse samples (5 mm) from the middle of the scar (infarct), infarct border (containing infarct and non-scarred limiting tissue) and from the septum non-infarcted wall (remote myocardium) of each slice were obtained, and snap-frozen in OCT, for histological and immunohistochemical evaluation.

Spatial transcriptomics using GeoMx DSP

OCT-embedded cardiac tissues harvested at 8 days (Short-term CO; n = 1) and 30 days (Long-term CO; n = 1) post-transplantation were cryosectioned and stained with fluorescently labeled antibodies targeting specific cellular markers: human nuclear antigen (HNA; Sigma, MAB1281), von Willebrand factor (vWF; Abcam, ab6994), cardiac troponin I (cTnI; Abcam, ab188877), and the nuclear dye SYTO83 (Thermo Fisher, S11364). Following staining and probe hybridization at 37 °C, slides were imaged using the GeoMx Digital Spatial Profiler (NanoString Technologies), yielding high-resolution fluorescence scans for spatial profiling.

Regions of interest (ROIs; 500 – 600 µm in diameter) were manually selected based on histological features and antibody staining patterns, comprising 19 ROIs from the Short-term CO tissue and 18 ROIs from the Long-term CO tissue. Within each ROI, areas of illumination (AOIs) were defined via morphological segmentation guided by HNA immunofluorescence to identify human-derived cells within the porcine cardiac tissue.

Transcriptomic profiling was performed using the Human Whole Transcriptome Atlas (WTA) panel. Spatially barcoded oligonucleotides were released from each AOI via UV illumination and collected into individual wells of a 96-well plate, as previously described (Merritt et al., 2020; https://doi.org/10.1038/s41587-020-0472-9). Barcode pools were subsequently hybridized, PCR-amplified, purified, and sequenced on an Illumina NextSeq 2000 platform. Raw FASTQ files were processed using the GeoMx DSP analysis pipeline, yielding digital count conversion (DCC) files and transcript count matrices for each ROI. Metadata annotations included timepoints (8 days, n = 19 ROIs; 30 days, n = 17 ROIs), enabling spatially resolved transcriptomic comparisons across conditions.

Data analysis and visualization were performed in R (v4.3.2) using RStudio (v2023.12.0.369) and the following packages: DESeq2, GSVA, ggplot2, dplyr, pheatmap, cowplot, limma, scran, scater, and tidyverse. Count matrices were filtered to retain genes with a row sum ≥ 10, and -log10 with P value advancement (padj). Differential expression analysis between the 30 day and 8-day conditions was performed using DESeq2. Gene regulations were classified via log2FoldChange > = 1 & padj < 0.05 ~ "up", log2FoldChange < = -1 & padj < 0.05 ~ "down", and TRUE ~ "not significant." Volcano plot was generated to visualize differentially expressed genes.

Gene set variation analysis (GSVA) was conducted on normalized expression values to assess enrichment differences in predefined gene sets (e.g., TGF-β, TGF-β inhibitor, and extracellular matrix remodelling pathways). Wilcoxon rank-sum tests were used to identify significantly ($P < 0.05$) different GSVA scores between the 8- and 30-day groups.

Results were visualized using heatmaps (top 50 differentially expressed genes), dot plots (selected cardiac marker genes across ROIs and time points), and violin plots (GSVA scores stratified by group). In violin plots, statistical significance was defined as $P < 0.05$.

SUPPLEMENTARY TABLES

Table supplementary S1 – S8 are excel sheets, which they are allocated in the Table supplementary folder named:

Table S1: hiPSC-Meso_Comparison-gene-analysis
Table S2: hiPSC-Mesodermal Ontology BP
Table S3: 96well-COs-Day20_positive-differentially-marker-genes
Table S4: 96well-COs-20_percentage_markers.markers
Table S5: 96well-COs-Day10&Day20_positive-differentially-marker-genes
Table S6: Bio-COs-Day20_positive-differentially-marker-genes
Table S7: Bio-COs-Day20_percentage_markers.
Table S8: GeoMx_Spatial-Transcriptomic_Pig-MI_8Days30Days__deseq2_all_genes_with_regulation_All-ROIs

Table S9: Composition of maturation medium (M-medium). The M-Medium had been optimized by Sarkawt Hamad, (2022), Marga-and-Walter-Boll Laboratory for Cardiac Tissue Engineering, University of Cologne, Cologne, Germany

Components	Company	Catalogue #	Quantity	Unite
**Advance DMEM:F12**	Gibco	12634-010	1	mL
**L-Ascorbic acid phosphate magnesium salt n-hydrate**	Wako, China	013-12061	100	µg
**Insulin**	Lilly	PZN-07242485	20	µg
**Transferrin**	Sigma-Aldrich	T3705-1G	5.1	µg
**Sodium selenite**	Sigma-Aldrich	S5261-10G	0.015	µg
**Heparin sodium salt**	Sigma-Aldrich	H3149-10KU	0.10	µg
**Recombinant human fibroblast growth factor-basic 154 a.a. (bFGF)**	Peprotech	100-18B	10	Ng
**Recombinant human vascular endothelial growth factor (VEGF)**	Peprotech	100-20	10	Ng
**3,3',5'-Triiodo-L-thyronine (rT3)**	Biomol	Cay17598-10	20	Ng
**Dexamethasone (Dexa)**	Sigma-Aldrich	D4902	200	Ng
**Oelic acid**	Sigma-Aldrich	1383	100	µM
**Palmic acid**	Sigma-Aldrich	90500	50	µM
**Creatine monohydrate**	Sigma-Aldrich	C3630	1.7	mM
**L-Carnitine hydrochloride**	Sigma-Aldrich	C0283	2	mM

**Taurine**	Sigma-Aldrich	Taurine	5	mM
**Calcium chloride**	Merk	A935382 948	1.5	mM
**L-Glutamine**	ThermoFisher Scientific	A2916801	2	mM
**2-Mercaptoethanol, 50 mM, 1000x**	Gibco	31350010	0.05	mM
**Penicillin–Streptomycin solution (Pen/Strep)**	ThermoFisher Scientific	15140-122	100	Units

Table S10: Standard single cell quality control metrics of scRNA-seq.

**NO.**	**Cell quality control information**	**CO-Day10**	**CO-Day20**
**1**	Estimated number of cells	5,999	4,351
**2**	Fraction reads in cells	65.62%	48.32%
**3**	Mean reads per cell	54,929	72,954
**4**	Median UMI per cell	4,023	11,506
**5**	Total genes	28,645	26,255
**6**	Median genes per cell	1,716	3,095
**7**	Saturation	66.47%	43.39%
**NO.**	**Threshold on mitochondrial percentage**	**Fraction of cells**	
**1**	> 5%	51.84%	65.27%
**2**	> 10%	28.02%	49.97%
**3**	> 15%	10.1%	41.16%
**4**	> 20%	6.02%	30.98%
**5**	> 50%	1.77%	3.49%

SUPPLEMENTARY FIGURES

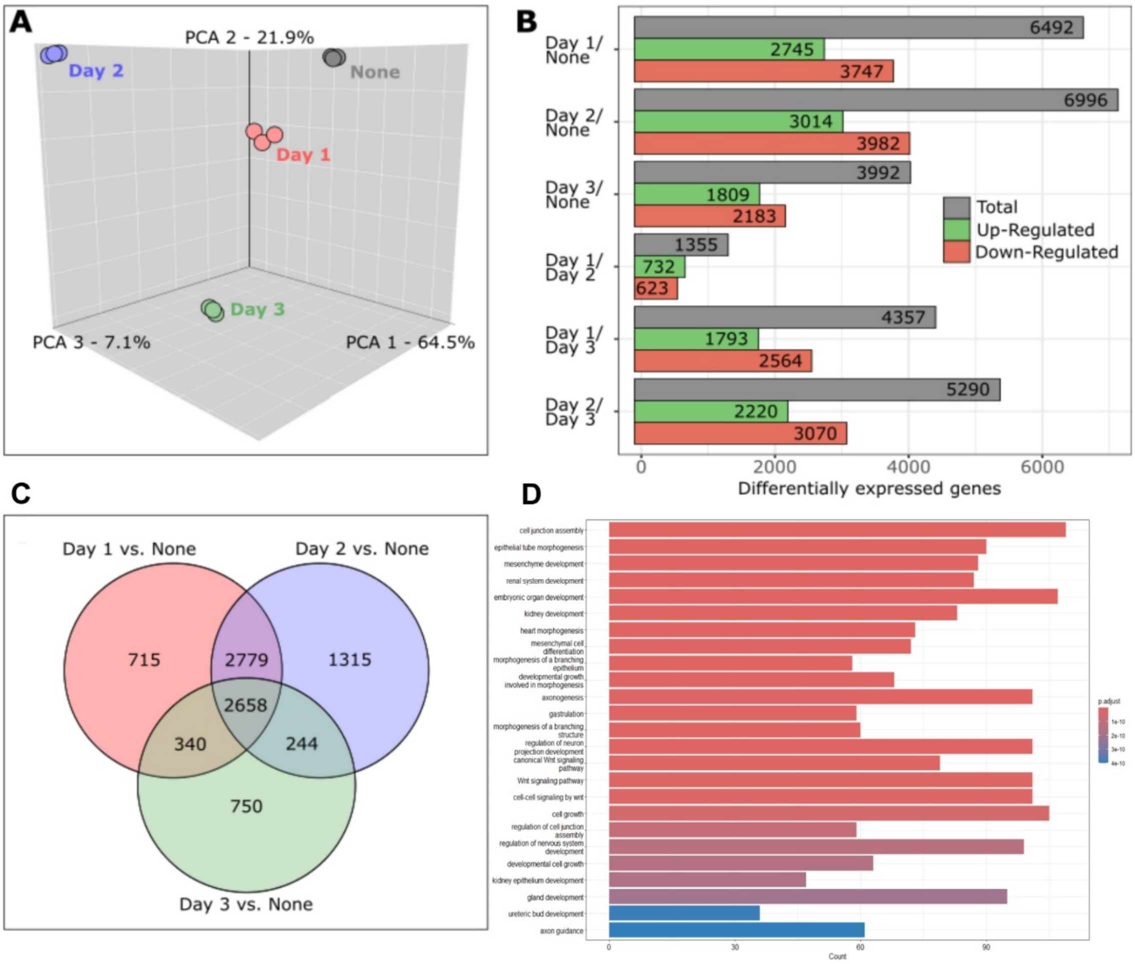

Figure S1: Microarray transcriptional analysis of mesodermal cells at day 1,2 and 3 after induction. (**A**) Principle compound analysis. (**B**) Comparison of > 2-fold up- and down-regulated transcripts as compared to non-induced control. (**C**) VENN diagram comparing numbers of individual and overlapping differentially regulated transcripts at different days of mesoderm induction. (**D**) Ontology bar graph of 24 top significantly positive biological pathways day 2 (induction) vs none (non-induction; control) of mesodermal cells.

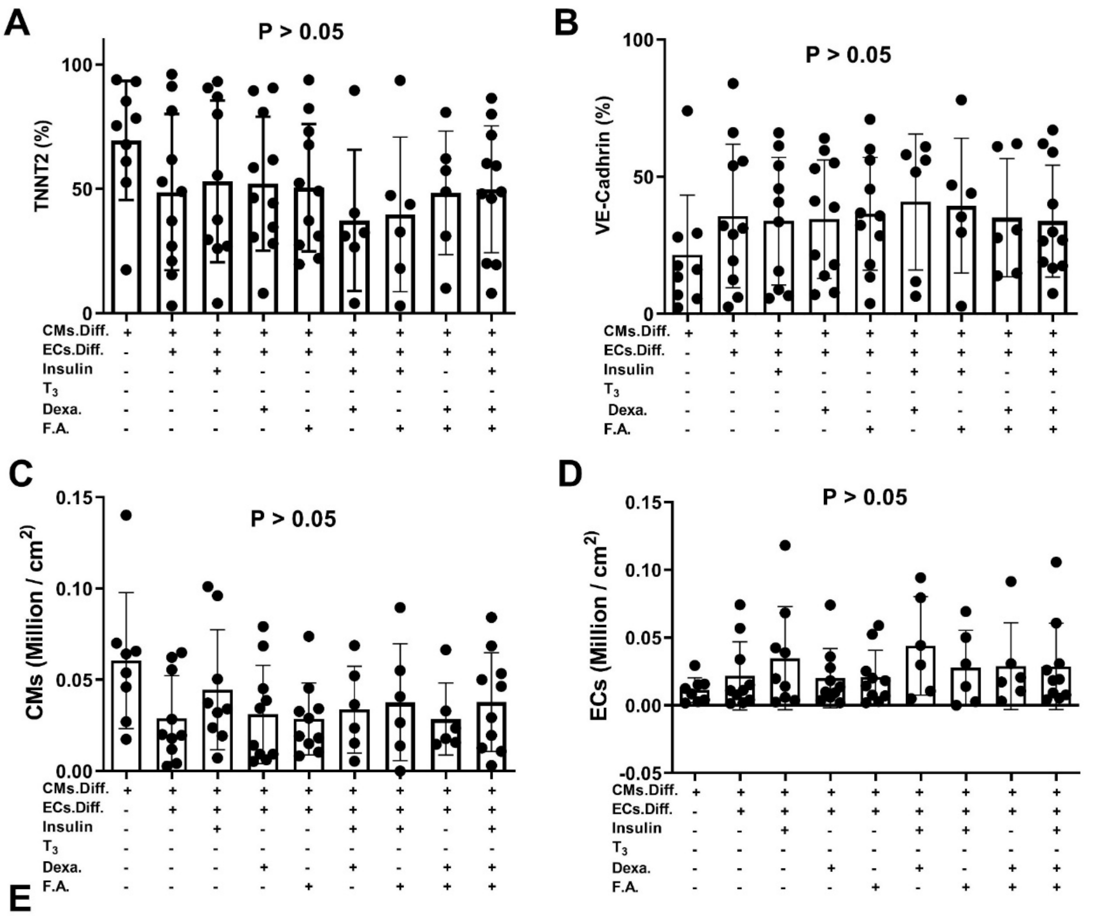

E

Descriptions		Control (hiPSC-CMs Diff)	Co.Diff	Co.Diff + Insulin	Co.Diff + Dexa.	Co.Diff + F.A.	Co.Diff + Insulin + Dexa.	Co.Diff + Insulin + F.A.	Co.Diff + Dexa. + F.A.	Co.Diff + Insulin + Dexa. + F.A.		
VE-Cadherin (%)	Mean	21.5	35.7	33.8	34.5	36.5	40.8	39.5	35.1	33.8	40.8	Max
	S.D	21.8	26.2	23.3	21.6	20.6	24.8	24.6	21.6	20.4	20.4	Min
	Actual confidence level	96.09%	98.83%	97.85%	98.83%	98.83%	96.88%	96.88%	96.88%	98.83%	98.83	Max
TNNT2 (%)	Mean	69.4	48.7	53.0	52.1	50.5	37.3	39.7	48.3	49.8	69.4	Max
	S.D	23.9	31.4	32.5	26.9	25.6	28.4	31.1	24.9	25.5	23.9	Min
	Actual confidence level	96.09%	98.83%	97.85%	98.83%	98.83%	96.88%	96.88%	96.88%	98.83%	98.83	Max
sample size (n)		9	11	10	11	11	6	6	6	11		
Number of green cells indicate of best		2	2	0	2	2	1	0	0	3		

Figure S2: Optimization of maturation medium composition. Flow cytometry analysis showing the percentage of cells positive for (**A**) cardiac troponin T (TNNT2), a cardiomyocyte marker, and (**B**) vascular endothelial cadherin (VE-cadherin), an endothelial cell marker. (**C, and D**) Total number of cardiomyocytes (**CMs; C**) and endothelial cells (**ECs; D**) per cm², calculated by subtracting the TNNT2 and VE-cadherin populations from the total cell count, respectively. (**E**) Summary table showing the mean, standard deviation (S.D) and actual confidence level for TNNT2$^+$ and VE-cadherin$^+$ cell percentages across each experimental group. The group yielding the highest number of green cells inside the table was considered optimal based on the highest mean, lowest S.D, and highest actual confidence level.

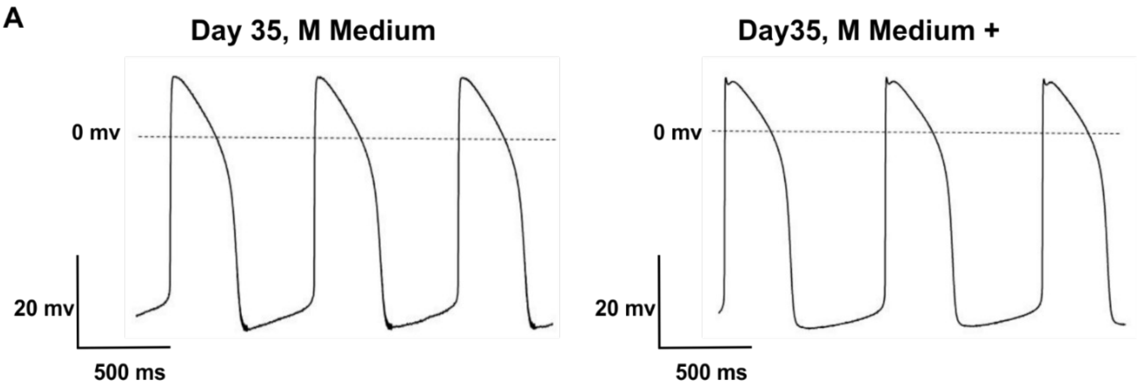

B

	Amplitude [mV]	MDP [mV]	APD50 [ms]	APD90 [ms]	APD 50/90 [%]	Vmax [V/s]	Frequenz [Hz]	n=
Ø	69.75±13.93	-55.23±9.11	350.60±90.47	390.47±89.80	89.18±2.66	6.63±4.83	1.8±0.3	2
T3	91.71±2.02	-71.60±1.1	517.31±34.80	556.76±33.31	92.74±0.81	13.90±2.02	1.14±0.12	5

t-test; p-value	Amplitude [mV]	MDP [mV]	APD50 [ms]	APD90 [ms]	APD 50/90 [%]	Vmax [V/s]	Frequenz [Hz]
Ø vs T3	0.035	0.248	0.078	0.072	0.127	0.147	0.057

Figure S3: Sharp electrode recording of CO action potentials without and with rT3 supplementation on day 35. (**A**) raw diagram, and (**B**) statistics of cardiac organoid electrical activity.

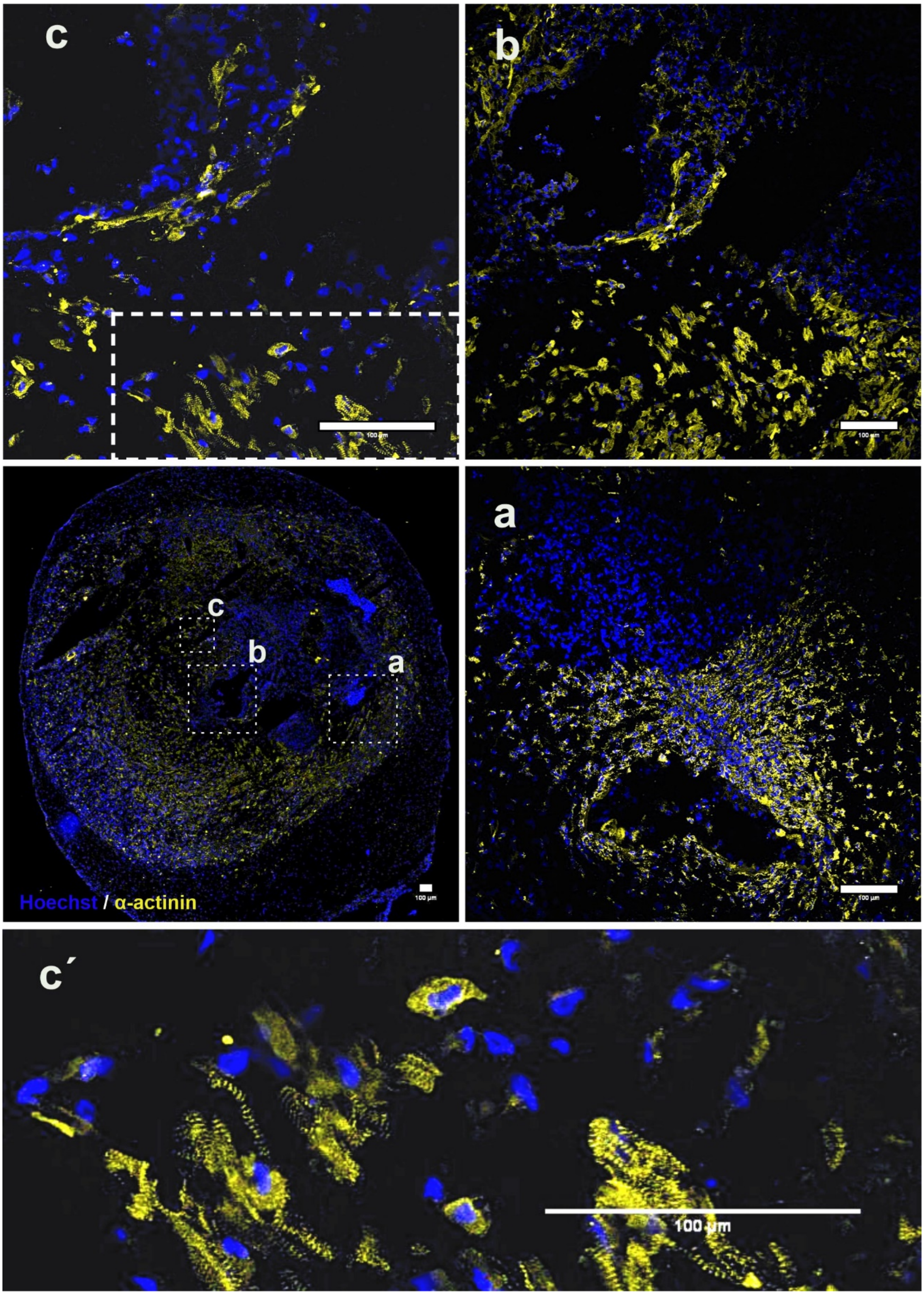

Figure S4: Immunostaining of fixed cardiac organoid slice at day 100.

The panels a, b and c represent higher power magnifications of the respective areas marked in the overview scan. The lower panel zooms into the lower part of region c illustrating the presence of intact sarcomeric structures in the inner core of the organoid. scale bar: 100 µm

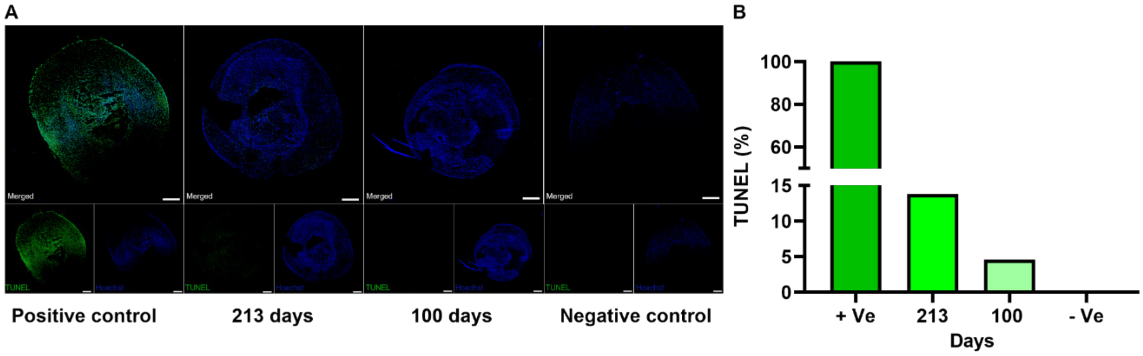

Figure S5: TUNNEL analysis of cardiac organoids at day 100 and 213. (**A**) confocal images. Scale bar: 100. (**B**) percentage of positive fluorescence of sliced TUNEL staining vs negative (-Ve) control unstained, and positive (+Ve) DNase treated slice prior stained for TUNEL, where call calculation normalized to maximal positive stained in +Ve group.

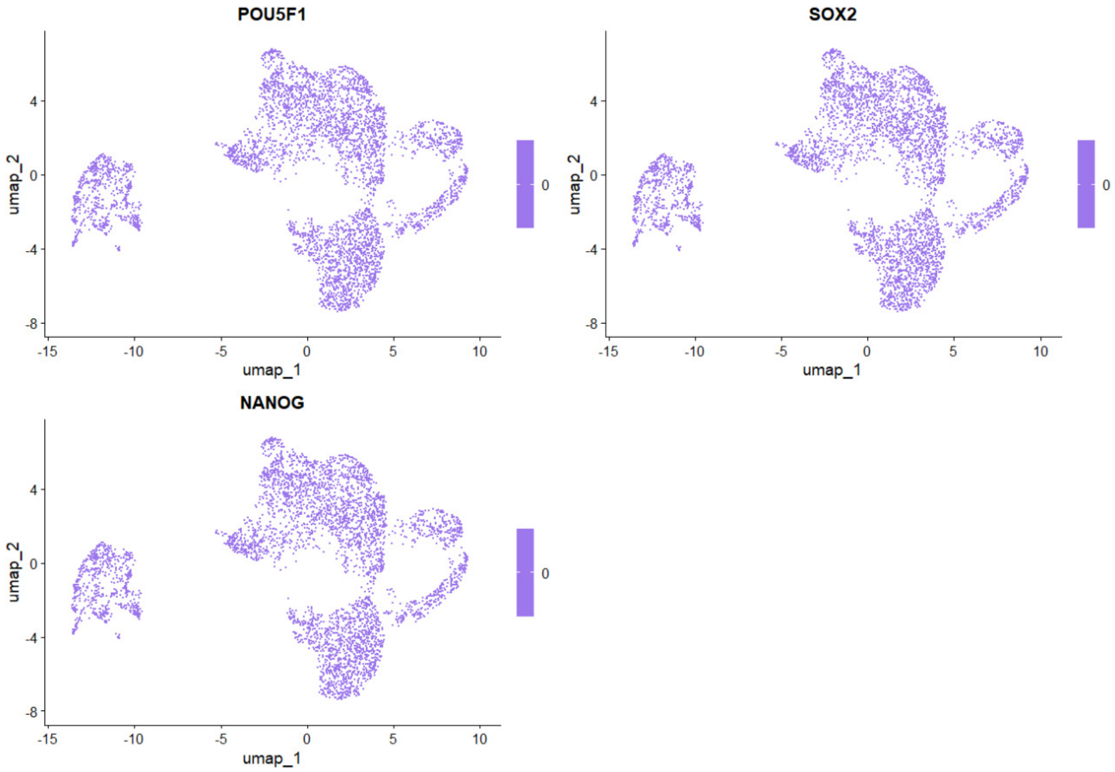

Figure S6: Single cell RNA sequencing of 96-well plates COs on day 20. The UMAP figure illustrates the absence of the expression of pluripotency markers on single cell level.

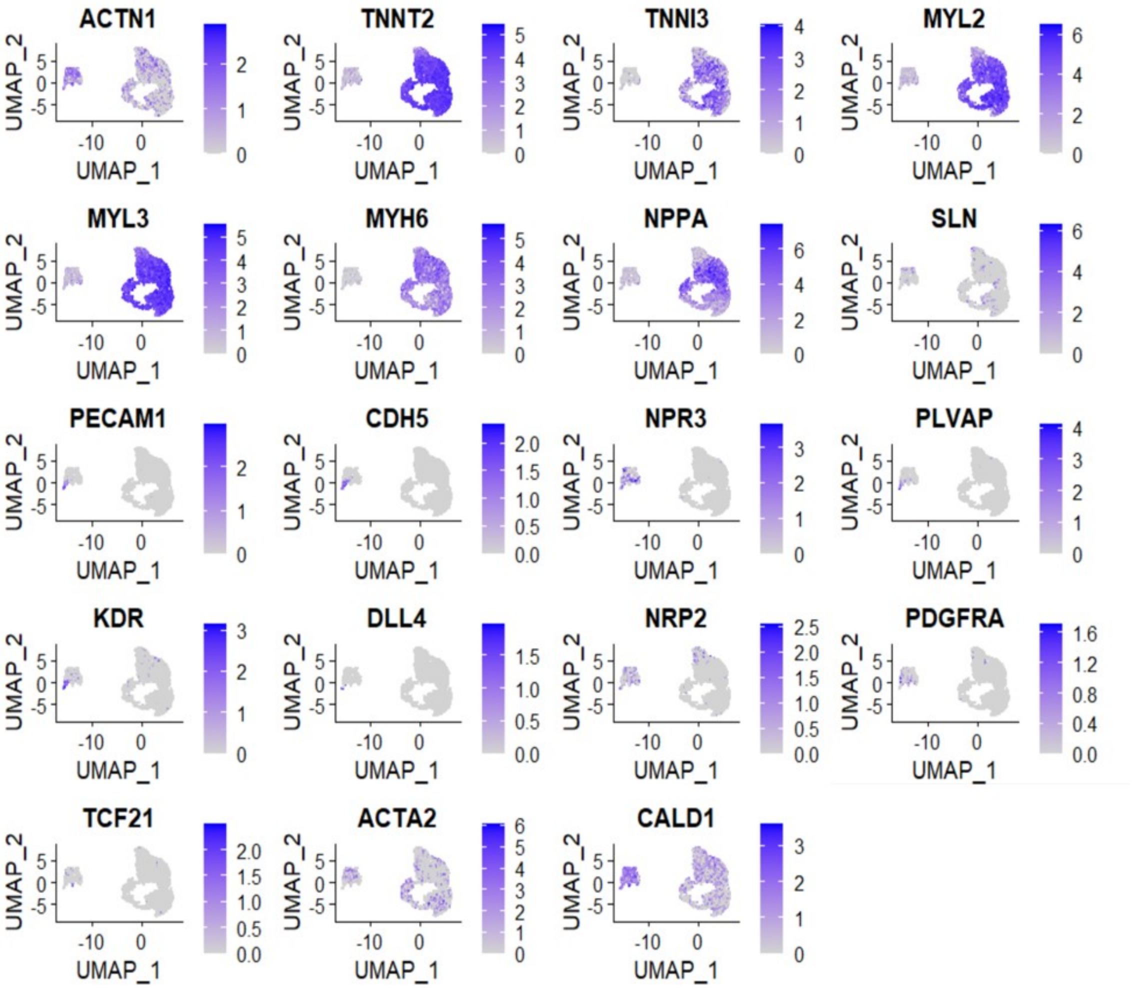

Figure S7: Single cell RNA sequencing of 96-well plates COs on day 20. The UMAP figure illustrates the expression of key cardiac cell marker genes on single cell level.

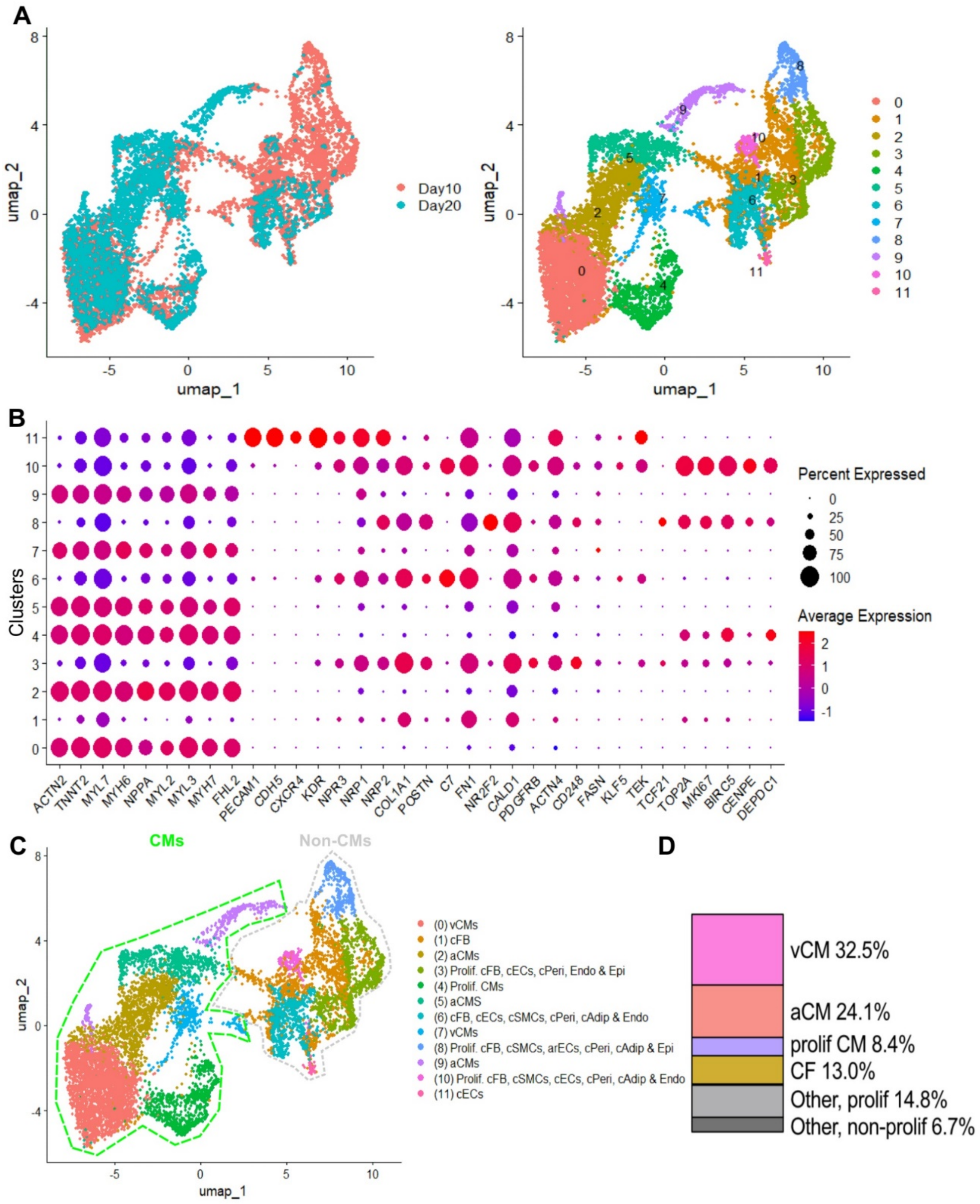

Figure S8: Integrated single cell RNA sequencing of COs from day 10 and 20. (**A**) sc-RNA-Seq data from day 10 and 20 of 96-well plate COs were merged, normalized, scaled, KNN applied to cluster integrated anchored data, UMAP plot revealed 12 distinct clusters, Wilcoxon test was applied for differentially expressed markers (genes) per cluster, and these genes were compared with PanglaoDB[21] and the Human Protein Atlas[22] data bases, and with published article to annotated the cluster identity. (**B**) Dotplot was used to compare our integrated sc-RNA-Seq data with published (see references in main text) cell markers. (**C**) clusters annotation. (**D**) After annotated each cluster to corresponding identity cell name, the percentage of cell clusters were analysed and annotated to the bar graph, where ventricular-like CMs (v-CMs = 32.5%), atrial-like CMs (a-CMs = 24.1%),

proliferating cardiomyocytes (prolif CM = 8.4%), cardiac fibroblast (CF = 13.0%), other proliferating cardiac cells (Other, prolif = 14.8%), and other non-proliferating cardiac cells (Other, non-prolif = 6.7%). There were no expression of the pluripotent stem cells and other organ cells identity e.g., gut markers.

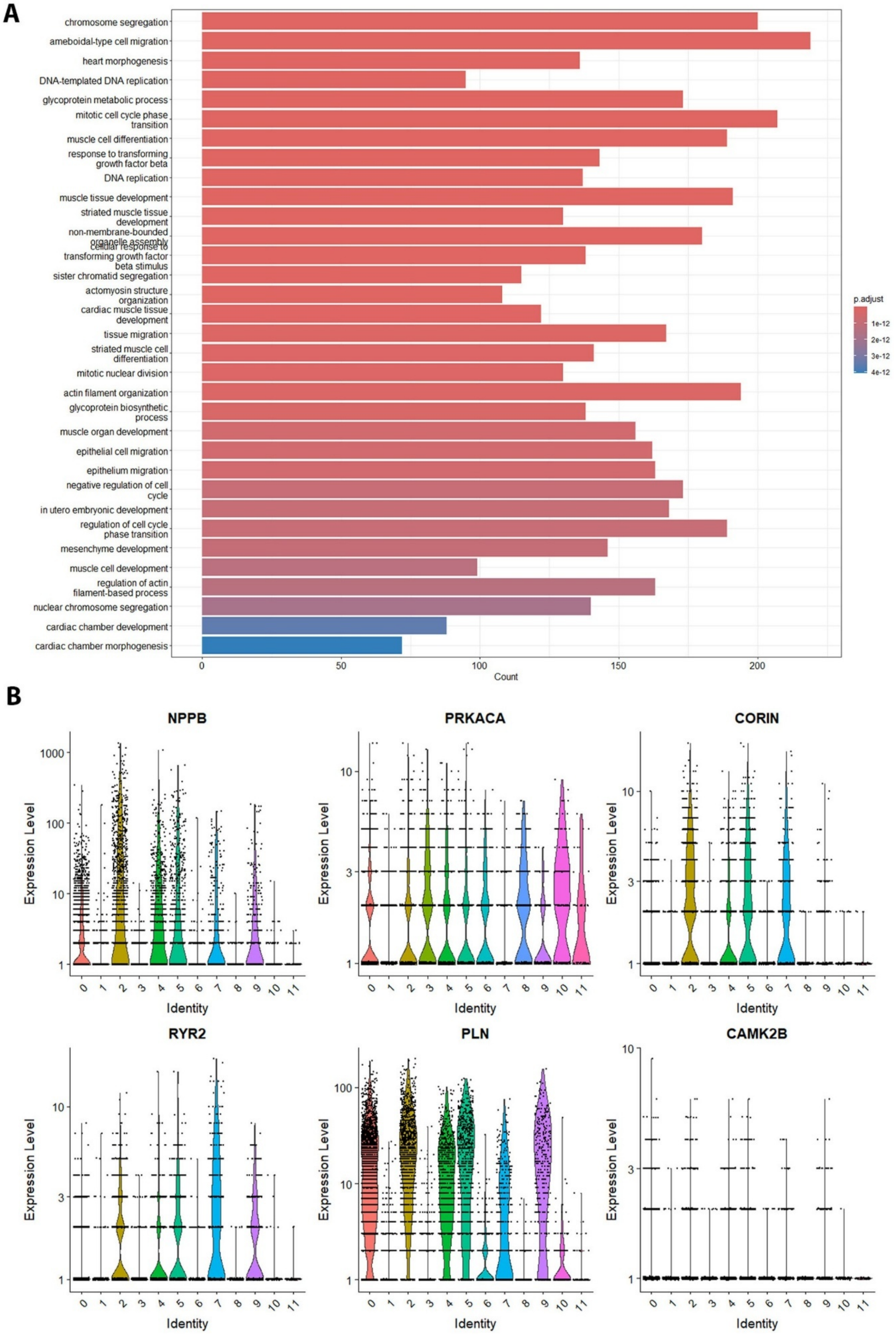
A
chromosome segregation
ameboidal-type cell migration
heart morphogenesis
DNA-templated DNA replication
glycoprotein metabolic process
mitotic cell cycle phase transition
muscle cell differentiation
response to transforming growth factor beta
DNA replication
muscle tissue development
striated muscle tissue development
non-membrane-bounded organelle assembly
cellular response to transforming growth factor beta stimulus
sister chromatid segregation
actomyosin structure organization
cardiac muscle tissue development
tissue migration
striated muscle cell differentiation
mitotic nuclear division
actin filament organization
glycoprotein biosynthetic process
muscle organ development
epithelial cell migration
epithelium migration
negative regulation of cell cycle
in utero embryonic development
regulation of cell cycle phase transition
mesenchyme development
muscle cell development
regulation of actin filament-based process
nuclear chromosome segregation
cardiac chamber development
cardiac chamber morphogenesis
0
50
100
150
200
Count
p.adjust
1e-12
2e-12
3e-12
4e-12
B
NPPB
PRKACA
CORIN
RYR2
PLN
CAMK2B
Expression Level
Identity
0 1 2 3 4 5 6 7 8 9 10 11
1000
100
10
1
3

Figure S9: Gene ontology (GO) analysis. (**A**) GO of single cell RNA sequencing data of significantly positive differentially gene expression of integrated scRNA COs at day 10 and 20 data. (**B**) Violin plot showing distribution of cells positive for NPPB, PRKACA, CORIN, RYR2, PLN, CAMK2B.

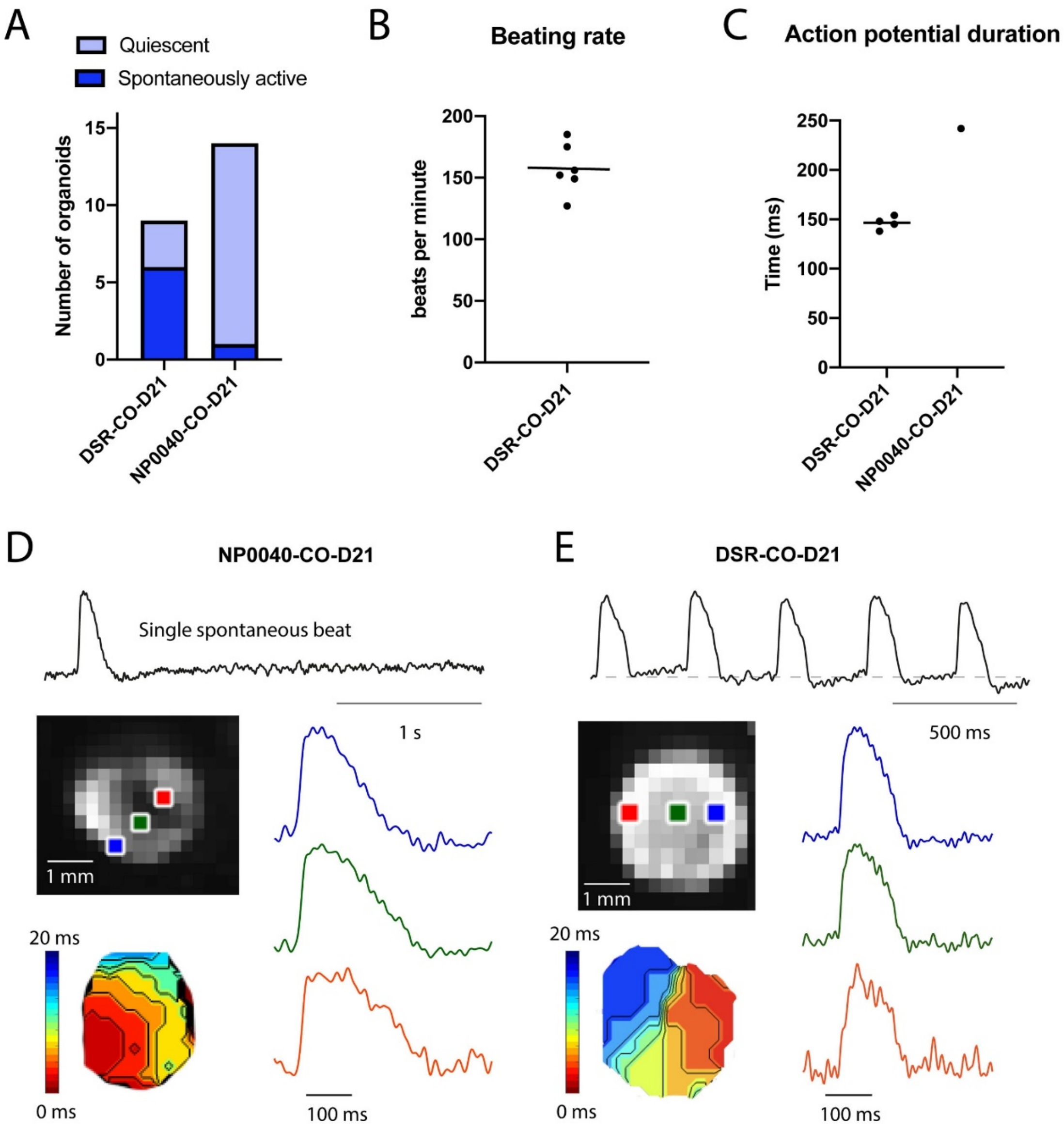

Figure S10. Optical mapping of CO at day 21. (**A**) Bar graph showing the number of organoids that were spontaneously active in batch DSR and NP0040. (**B**) The average beating rate of the organoids of batch DSR. (**C**) Graph showing the average action potential duration in both batch DSR and NP0040. (**D**) The upper trace shows a single optical action potential recorded from the cardiac organoids from batch NP0040. The three optical action potentials in blue, green and orange correspond to the location shown in the fluorescent photograph of the cardiac organoid. The map in the left bottom illustrates the activation sequence during the spontaneous beat. (**E**) The upper trace shows a single optical action potential recorded from the cardiac organoids from batch DSR. The three optical action potentials in blue, green and orange correspond to the location shown in the

fluorescent photograph of the cardiac organoid. The map in the left bottom illustrates the activation sequence during the spontaneous beat.

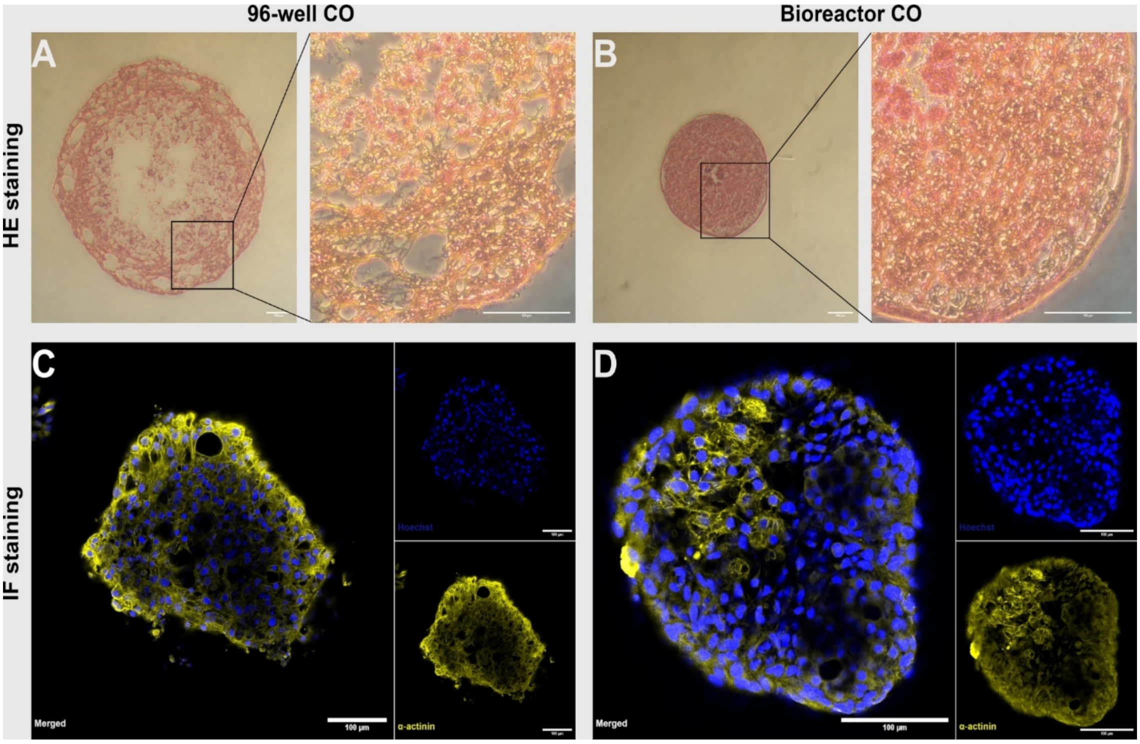

Figure S11: Hematoxylin and eosin staining of day 20 COs produced in 96-wells or in stirred bioreactor culture. (A, and B) A comparison of COs generated in 96-well plates and cultivated on a suspension shaker inside an incubator on orbital shaker from day 0 and COs generated inside a stirred tank bioreactor confirms the loose inner structure of the COs with small cavities. **(C, and D)**, where 96-well COs (**A, and C)**, and Bioreactor COs **(B, and D).** The respective images of these COs after immunofluorescence staining against α-actinin (yellow) and nuclei counterstained with Hoechst 33342 (blue). Scale bars = 100 µm.

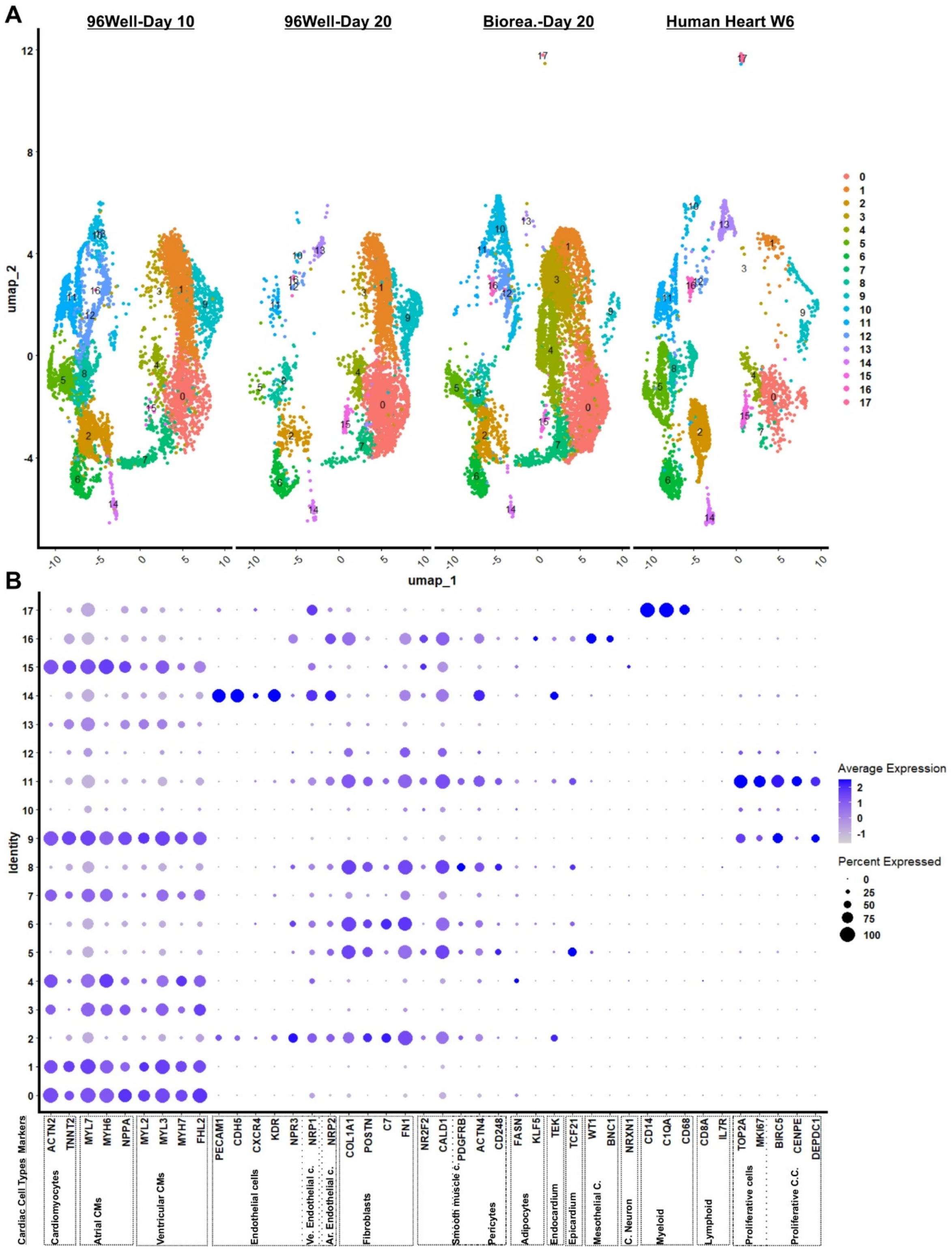

Figure S12. Integrated analysis of 96 well and bioreactor generated COs with human heart week 6. (**A**) cluster analysis of single cell sequencing data of COs (day 10 and day 20) generated on 96 well plates and cultured in suspension after day 0 in a shaking incubator compared to COs generated in a stirred suspension bioreactor (day 20) and human fetal heart at week 6 of gestation (HHW6). (**B**) dot plot and marker analysis of the clusters for the expression of cell type-specific marker genes.

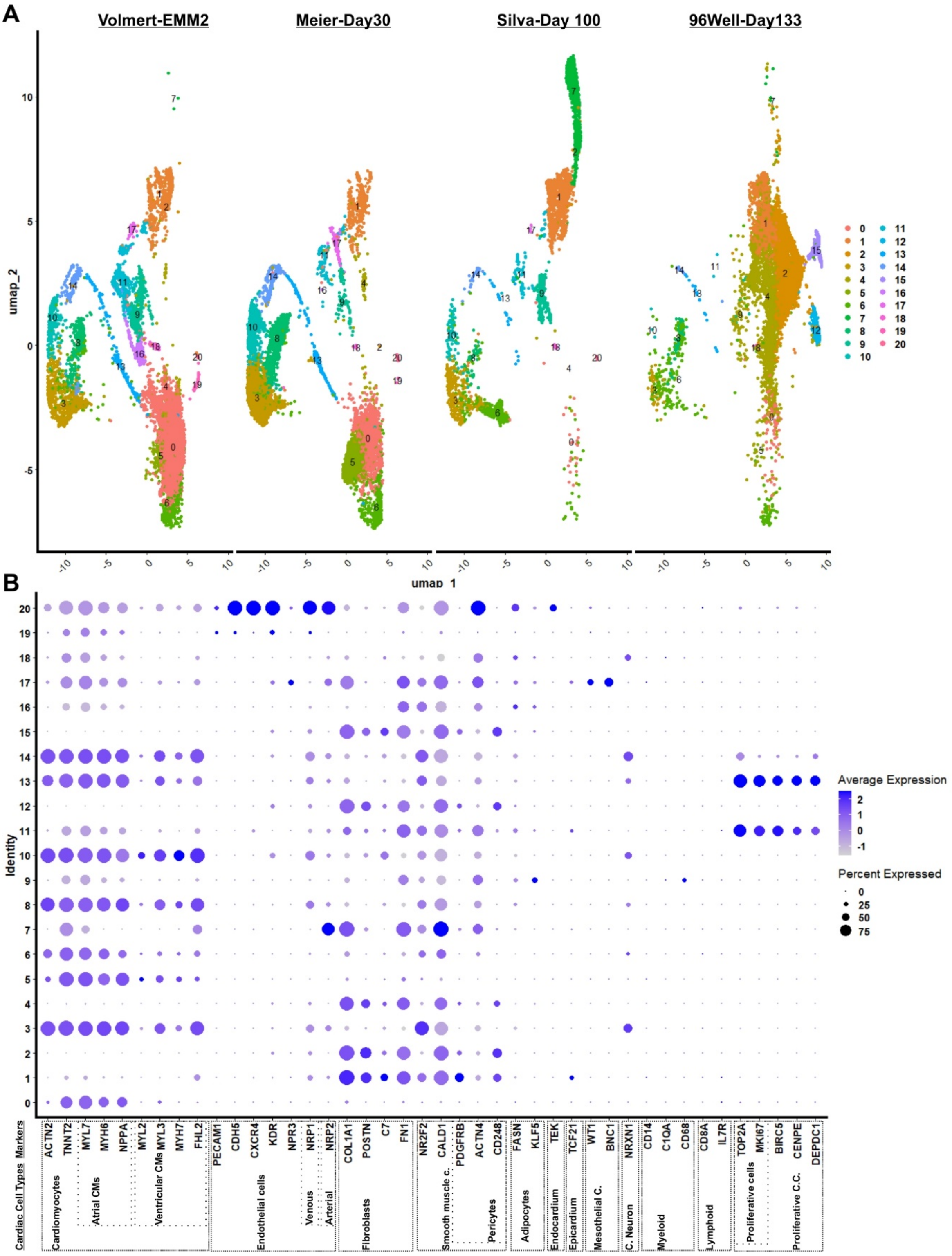

Figure S13. Integrated analysis of published single cell RNA sequencing datasets from 3 different sources and COs at day 133. (**A**) Cluster analysis of cardiac organoids based on published data from 3 different groups collected at day 30 or day 100 of differentiation and cardiac organoids generated by the protocol described here and collected at day 133. (**B**) Analysis of the clusters for the expression of cell type-specific marker genes.

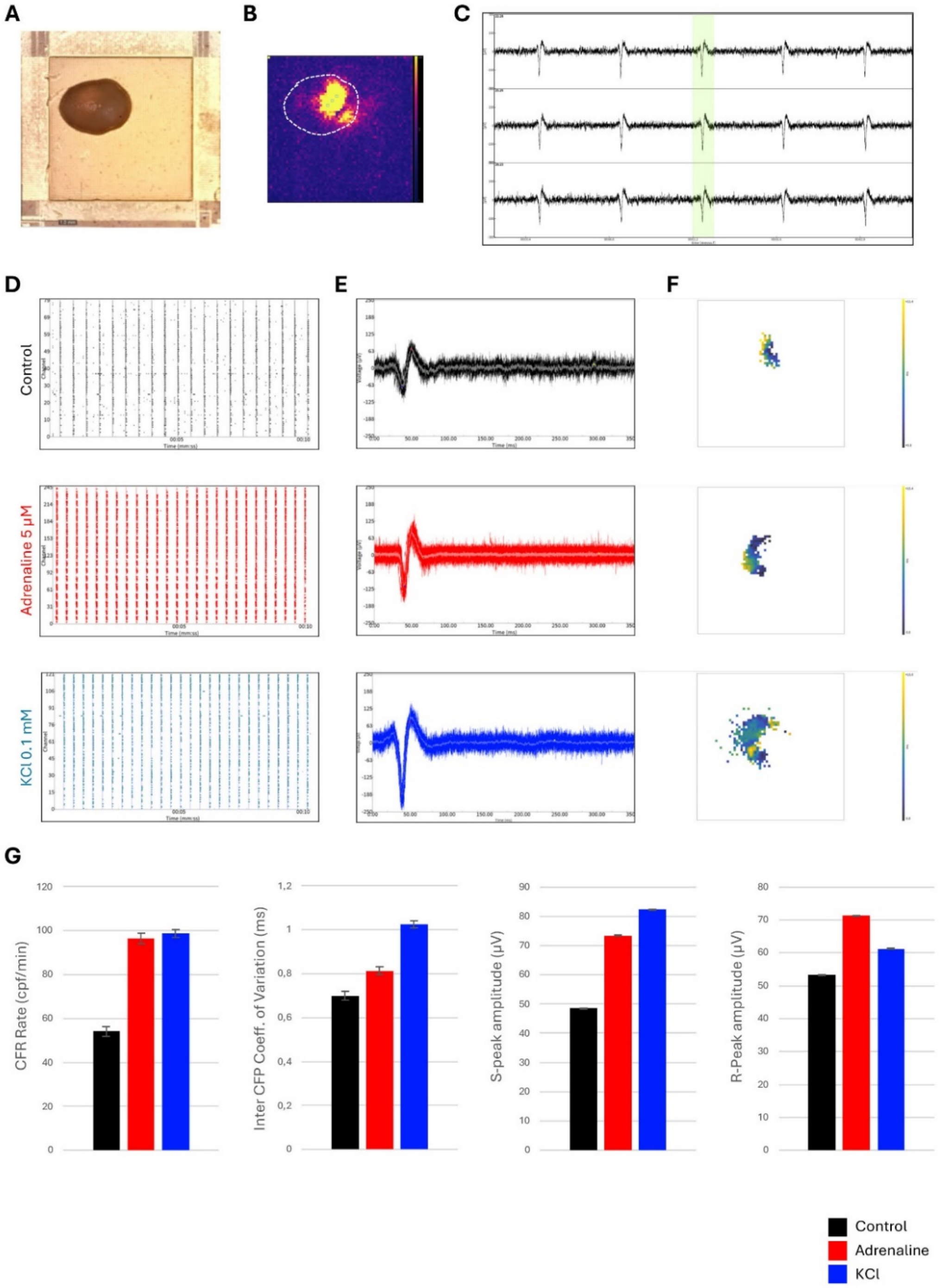

Figure S14. Electrocardiographic and drug response analysis of the COs. (**A**) Representative CO placed on a high-density microelectrode array (HD-MEA) and (**B**) activity map of the CO. Each pixel corresponds to the peak-to-peak amplitude of the raw signal recorded from a single electrode, providing a detailed overview of the organoid's electrical activity. (**C**) Example voltage traces from three electrodes recorded in a separate cardiac organoid. These traces illustrate spontaneous cardiac field potentials (CFPs) and serve as a baseline reference for pharmacological modulation in (**D - G**). The green shading indicates the data window used for computing the activity map. (**D**) Raster plots depicting firing activity of active electrodes (detecting CFPs with amplitudes > 100 µV) under

control conditions (black), after 5 μM adrenaline (red), and following 0.1 mM KCl (blue), demonstrating changes in beating regularity and frequency. (**E**) Representative CFP waveforms under the three conditions (control, adrenaline, KCl), with Q, R, S, and T peaks indicated. Notably, adrenaline and KCl increase the S-peak amplitude. (**F**) Propagation maps showing how electrical signals propagate under each condition, revealing KCl-induced conduction disturbances, including a reversal in the propagation direction. (**G**) Bar graphs displaying CFP rate, inter-CFP coefficient of variation, S-peak amplitude, and R-peak amplitude under control (black), adrenaline (red), and KCl (blue) conditions. The data represent the mean ± standard deviation.

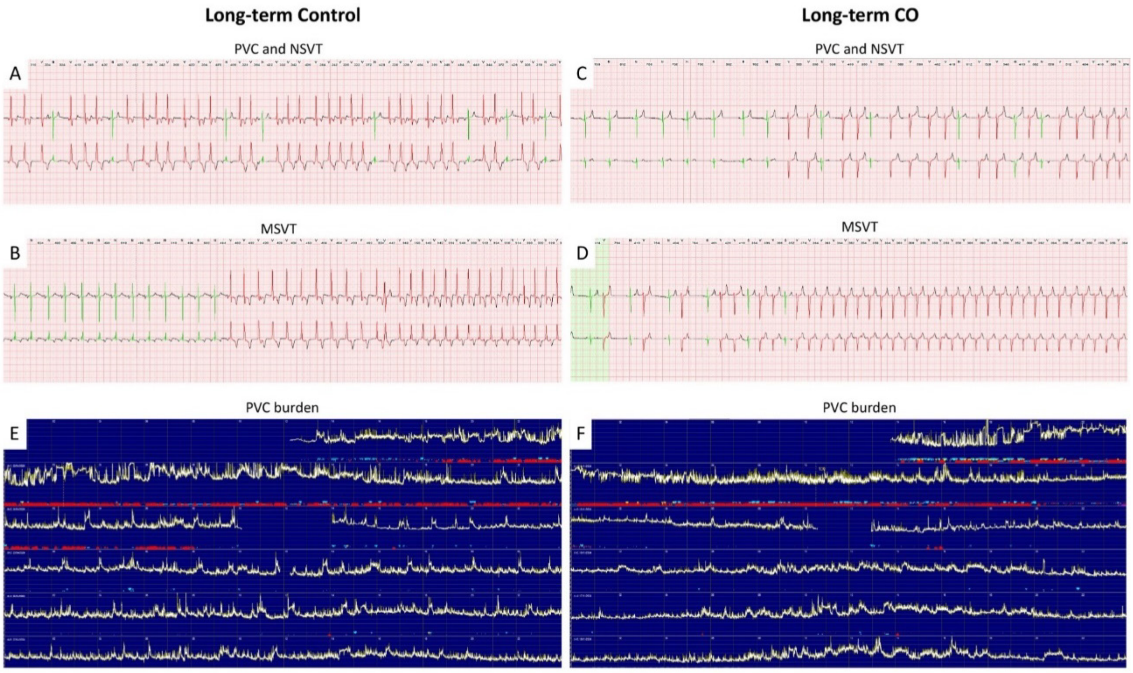

Figure S15. ECG-Holter monitoring. (**A, B**) Representative examples of ventricular arrhythmias of Long-term Control and (**C, D**) CO animals. (**A, C**) Both groups presented with high burden of PVC and NSVT and (**B, D**) MSVT at early phase after surgery (< 48h). Note the abrupt decline at 40 and 30 hours after surgery in (**E**) Long-term Control and (**F**) CO animals. Abnormal ventricular activity is marked in red.

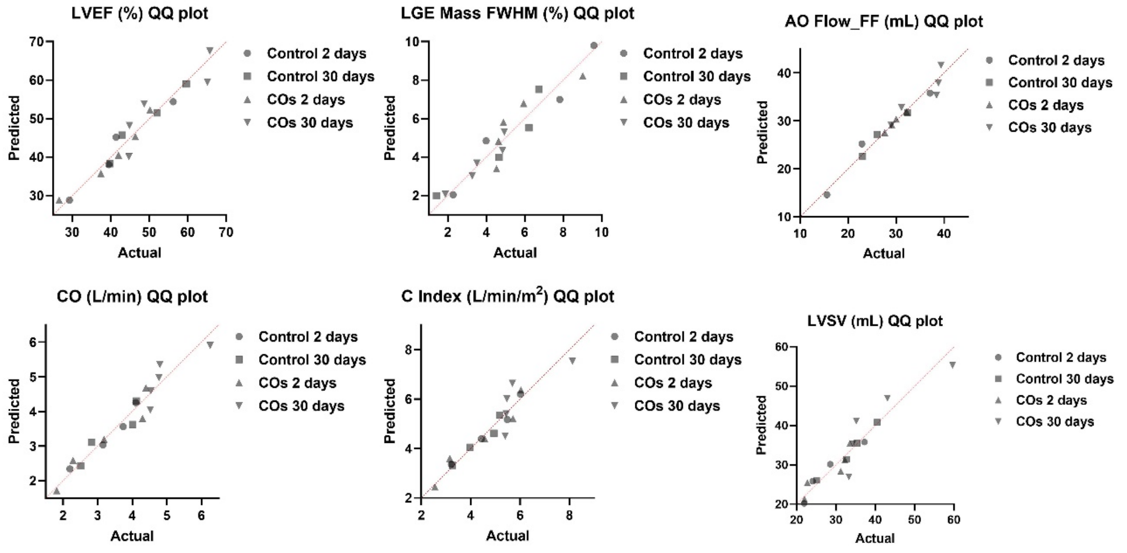

Figure S16: Q-Q plots of in vivo cardiac magnetic resonance imaging parameters.

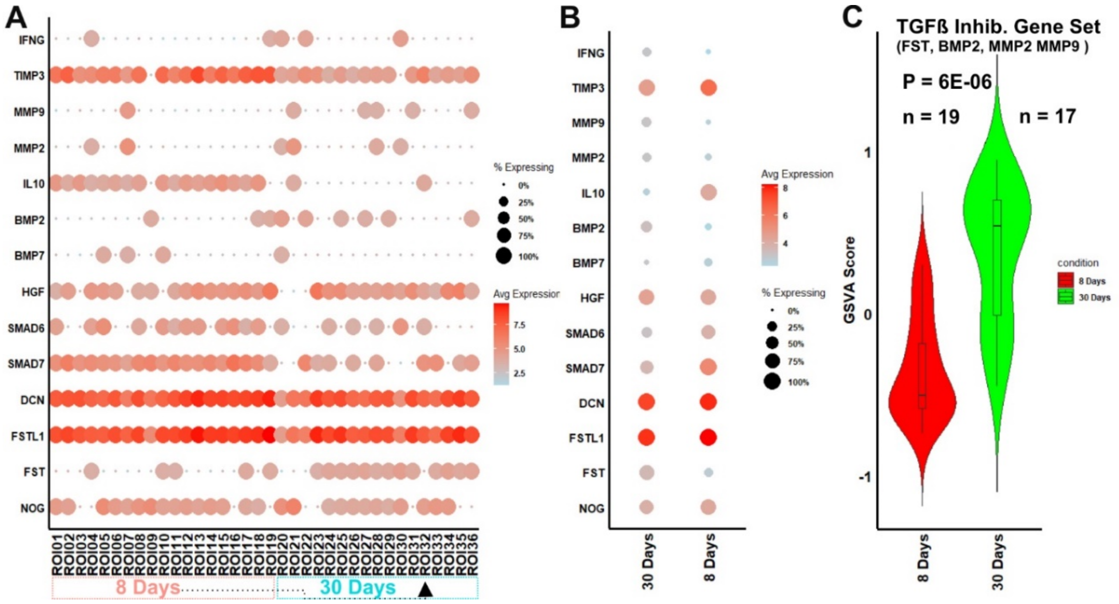

Figure S17. GeoMx spatial transcriptomics of porcine cardiac tissue at 8 and 30 days post-transplantation. Expression and pathway analysis of transforming growth factor-β (TGF-β) related inhibitory genes in porcine cardiac tissue following xenotransplantation. (**A**) Dot plot showing the expression of key genes associated with inhibition of TGF-β signaling, fibrosis regulation, and extracellular matrix remodeling across regions of interest (ROIs; a complete area considered as a replication, where ROI32 was part of 8 days group) in porcine cardiac tissue at 8 and 30 days post-transplantation. Genes include IFNG (interferon gamma), TIMP3 (tissue inhibitor of metalloproteinases 3), MMP9 (matrix metallopeptidase 9), MMP2 (matrix metallopeptidase 2), IL10

(interleukin 10), BMP2 (bone morphogenetic protein 2), BMP7 (bone morphogenetic protein 7), HGF (hepatocyte growth factor), SMAD6 (SMAD family member 6), SMAD7 (SMAD family member 7), DCN (decorin), FSTL1 (follistatin-like 1), FST (follistatin), and NOG (noggin). Dot size represents the percentage of ROIs expressing each gene; color scale reflects average gene expression intensity. (**B**) Summary dot plot comparing the average expression of selected TGF-β–modulatory genes between the 8-day and 30-day time points, showing increased expression of FST, BMP2, MMP2, and MMP9, and related inhibitors at 30 days. (**C**) Gene set variation analysis (GSVA) scores for a TGF-β inhibitory gene set composed of FST (follistatin), BMP2 (bone morphogenetic protein 2), MMP2 (matrix metallopeptidase 2), and MMP9 (matrix metallopeptidase 9), comparing 17 ROIs from 30 days vs 19 from 8 days. Significantly elevated pathway activity was observed in 30-day samples relative to 8-day samples (Wilcoxon rank-sum test, $P = 6 \times 10^{-6}$).
